# evALLution: making basic evolution concepts accessible to people with visual impairment through a multisensory tree of life

**DOI:** 10.1186/s12052-021-00143-1

**Published:** 2021-03-11

**Authors:** Telma G. Laurentino, Marisa Xavier, Fabrizia Ronco, Francisco Pina-Martins, Iolanda Domingues, Bruno Penha, Marta Dias, Alexandra de Sousa, Tiago Carrilho, Leonor R. Rodrigues, Carlota Pinheiro, Daniela Rato, Duarte Balata, Gonçalo Ayala-Botto, Margarida Matos, Maria Campelo, Rafael Botelho

**Affiliations:** 1 Zoology, University of Basel, Basel, Switzerland; 2 Department of Environmental Science, Policy, and Management, University of California, Berkeley, CA 94720 USA; 3 Mariza Xavier Design, Lisbon, Portugal; 4 cE3c-Centre for Ecology, Evolution and Environmental Changes, Faculdade de Ciências, Universidade de Lisboa (ULisboa), Campo Grande, 1749-016 Lisbon, Portugal; 5 College of Life and Environmental Sciences, University of Exeter, Exeter, UK; 6 Centre for Health and Cognition, Bath Spa University, Bath, UK; 7 Crossmodal Cognition Laboratory, University of Bath, Bath, UK; 8 Centro Pedagógico Do Jardim Zoológico de Lisboa, Lisbon, Portugal; 9 Tutisfore, Lisbon, Portugal

**Keywords:** Blind, Visual impairment, Evolution, Multi-sensory, Touch, Inclusive outreach, Accessibility

## Abstract

**Background:**

People with visual impairment have benefitted from recent developments of assistive technology that aim to decrease socio-economic inequality. However, access to post-secondary education is still extremelly challenging, especially for scientific areas. The under representation of people with visual impairment in the evolution research community is connected with the vision-based communication of evolutionary biology knowledge and the accompanying lack of multisensory alternatives for learning.

**Results:**

Here, we describe the development of an inclusive outreach activity based on a multisensory phylogeny representing 20 taxonomic groups. We provide a tool kit of materials and ideas that allow both the replication of this activity and the adaptation of others, to include people with visual impairment. Furthermore, we provide activity evaluation data, a discussion of the lessons learned and an inclusive description of all figures and visual data presented.

The presented baseline data show that people with visual impairment indeed have lack of access to education but are interested in and apt to understand evolutionary biology concepts and predict evolutionary change when education is inclusive.

**Conclusions:**

We show that, with creative investment, basic evolutionary knowledge is perfectly possible to be transmitted through multisensory activities, which everyone can benefit from. Ultimately, we hope this case study will provide a baseline for future initiatives and a more inclusive outreach community.

## Background

An estimated 36 million people worldwide are blind. Additionally, 217 million people have moderate or severe vision impairment (statistics for 2015)—these numbers are estimated to increase due to aging and diet related causes (Bourne et al. 2017). Vision is one of the dominant senses for information acquisition in humans. Thus, the inability to see is often associated with socio-economic inequity and limited access to education (Eurostat 2015; EU-SILC UDB (2014)). Less than half (44%) of the population with visual disability enrolls in post-secondary education and only 18% graduate (data from 2005 for USA population; (Newman et al. 2010). Reduced vision and blindness become a physical barrier to the individual’s learning experience due to the lack of multisensory alternatives to widespread learning activities (Salleh and Zainal 2010). Such lack of accessibility to knowledge is highly present in the fields of science, technology, engineering and mathematics (STEM) where instruction relies heavily on graphically conveyed information.

Efforts to develop assistive technology and promote the inclusive education of people with visual impairment are significant in some scientific areas (Cryer 2013), like chemistry (e.g. Fantin et al. 2016; Garrido-Escudero 2013; Supalo et al. 2008; Supalo and Kennedy 2014) and physics (e.g. Arcand et al. 2019; Ediyanto and Kawai 2019). However, the biological sciences seem to lag behind (but check Jones et al. 2006). More specifically, the teaching of evolution relies on visual media as its primary communication mechanism for conceptual understanding. Classical evolution case studies commonly used in formal education and outreach activities–such as beak morphology evolution in Darwin’s finches (Grant and Grant 2002) or the industrial melanic peppered-moth selection (Cook et al. 2012)—are based on phenotype-environment associations and selective forces that demand a priori understanding of variability in shape, size and colour traits. All of these are visual characteristics of information, which are hard to grasp by people with reduced vision and inaccessible to people born blind.

The understanding of natural patterns is further compromised by the fact that the research of such case studies is communicated through 2-dimensional tables, plots and diagrams, available solely on screen or paper, all of which are particularly challenging to access for those with severe vision impairment (Karshmer and Bledsoe 2002; McCarthy and Shevlin 2017). Consequently, although teenagers with visual impairment show high interest in STEM areas, their motivation to pursue a carrier in such areas is reduced by the barriers felt while trying to learn (Bell and Silverman 2018). In fact, only one percent of STEM doctorate recipients has any sort of reported disability (data for the U.S. population; (Moon et al. 2012).

It is thus clear that there is an urgent need for improved accessibility to scientific knowledge in order to promote equity in education and a more diverse and inclusive scientific community. Outreach activities that transmit knowledge with a multisensory approach can be an important first step towards that end (Pérez-Montero 2019) and are known to benefit both the audience and the scientific community (Clark et al. 2016).

Our project aims at contributing to equity in accessibility to evolutionary biology knowledge by eliminating physical barriers to the understanding of the basic mechanisms of evolution and the resulting biodiversity pattern.

We here describe the development of a multisensory phylogeny, designed as an introduction to basic concepts in evolution for people with severe visual impairments.

We provide a tool kit that enables the repeatability of this activity together with guidelines that can be adapted and applied to several other outreach initiatives. We propose a two-step rationale to approach inclusive evolution teaching: The public needs to first (I) experience the pattern of biodiversity so that then we can (II) discuss the processes that led to such diversification. In addition to activity design, baseline data on the evaluation of the activity are presented.

## Methods

### Reproducibility framework

Touch as the main sense of communication raises challenges: natural history collections are usually too unique or fragile to be freely manipulated, live specimens pose animal welfare concerns, and commercially available models can be inaccurate and do not portray the real and detailed textures and patterns of biodiversity.

Our activity was developed during a whole year, which encompassed a great deal of communication with the blind community, psychologists, science communicators, museologists, evolutionary biologists and pedagogic institutions.

**Fig. 1:**
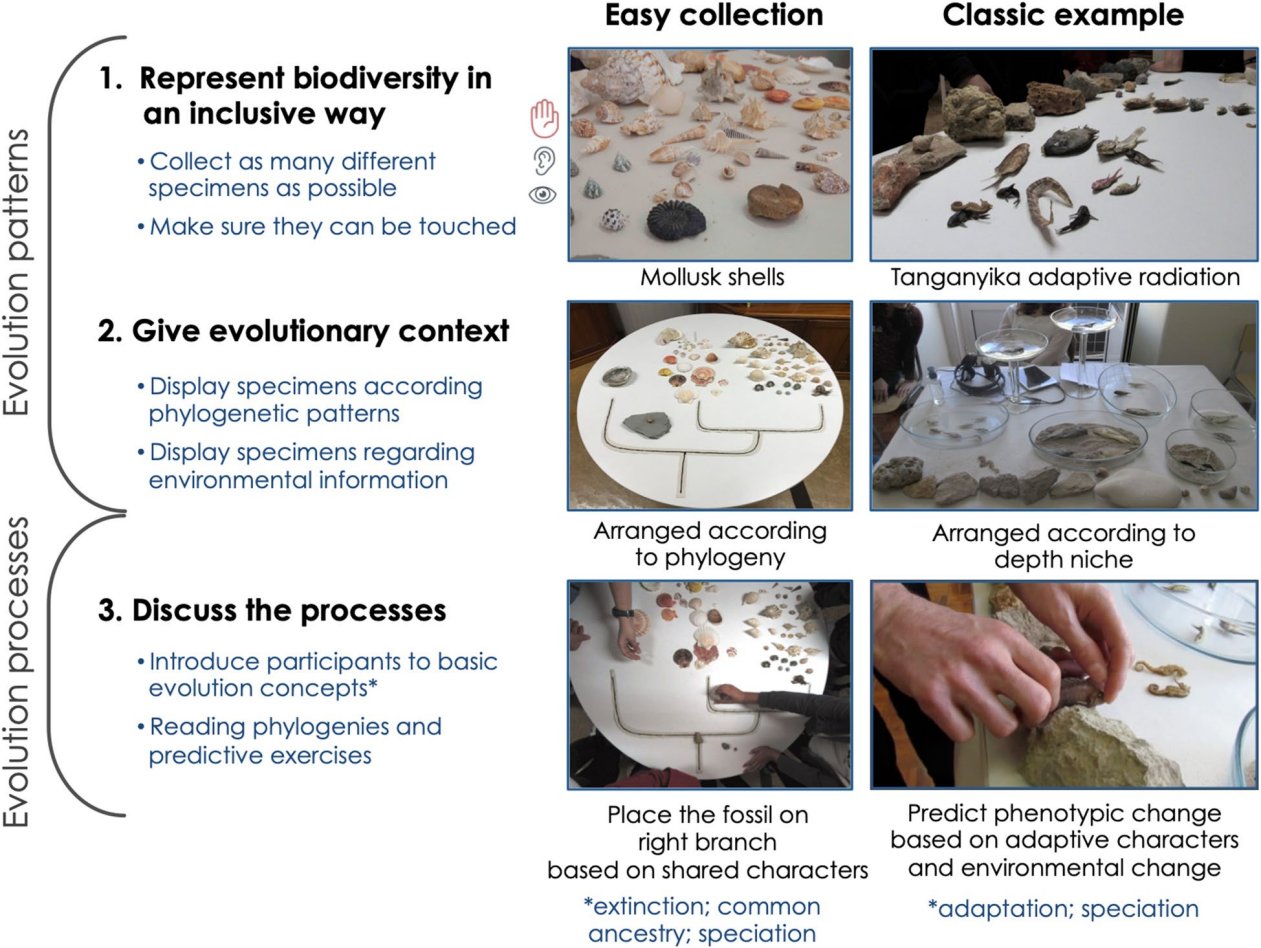
Basic framework to construct evolution outreach activities inclusive for people with blindness. Two types of materials are depicted: a collection of general easy access (Mollusk shells) and a classic evolution textbook example of adaptive speciation (the adaptive radiation of cichlids in the Tanganyika lake). Following this framework, all gathered materials are able to be adapted for inclusive outreach activities, independently of amount of branches represented and logistics. The pictures of the mollusk phylogeny on the round table are from a second activity, at an inclusive school, where the available room was significantly smaller than the original 125 m^2^ space, thus more activities per floor-phylogeny branch (8 instead of 21) were included

Based on the experience acquired from that process and the results of our activity, we here provide a theoretical framework to organize similar activities (Fig. 1).

There are three main steps to conveying evolution’s patterns and processes to a public with visual disability: (1) make biodiversity accessible, (2) give an evolutionary and ecological context to the displayed biodiversity and (3) discuss the processes and evolutionary forces through which that biodiversity evolved.

1. First, we want to convey biodiversity as the resulting pattern of evolution. Sighted people can easily grasp the diversity of living beings and ecosystems through images; in order to include people with blindness we have to represent as much biodiversity as possible in an inclusive way. For this, communication with the blind community and creativity are essential. Sighted people tend to use auditory explanatory cues to convey information. However, this does not only create an immediate barrier for people with hearing disabilities, but our consultant from the blind community was quick to explain that touch had to be the main sense used. He made it clear to us with the following example: “If you tell me that a zebra is a horse with black and white stripes, and I was born blind, I will most likely not know what horses look like; stripes and colors might also be concepts that I do not understand”. Thus, all collected materials should have the potential to be touched and then accompanied with auditory instruction by the teaching volunteers. To include people with hearing loss, the instruction should also be translated into sign language.
  Collections such as Mollusks’ shells and fossils are easily obtainable in great number and diversity–it was one of the resources more museums were willing to lend. The shape diversity within the phylum is accessible through touch, but the diversity of color patterns needs to be translated to haptic cues. When posed with such challenges we recurrently used hot glue to trace the pattern we wanted to be accessible. Hot glue is a great resource to turn 2D patterns into 3D (see butterfly wing models in Additional file 1: Fig. S1f; and mollusks table phylogeny in Fig. 1).
  Another easily accessible taxon is plants. Gardening centers in general have a great diversity of worldwide plants that can be easily sourced. We found that, despite it being the most familiar, participants responded very well to the plant branch, spending a lot of time there, and were excited by learning about plants’ adaptations to specific environmental conditions.

2. Then, to approach biodiversity and evolution, the collected materials should be displayed in evolutionary context. For this, the specimens representative of the main branches can be displayed in several informative ways: (a) following the phylogeny of the species or orders depicted (e.g., mollusks organized as Monoplacophora, Bivalvia and Gastropoda; Fig. 1, left); (b) according to niche within an environment (e.g., Lake Tanganyika cichlids were arranged according to the depth (height) and substrate (sand, stones) they inhabit in the lake; Fig. 1, right); or (c) across different environments (e.g., plants were organized by climate regime including desert, tropical forest, Mediterranean forest and Taiga (Additional file 1: Fig. S1 t–w).
  These meaningful displays, together with oral pedagogic information, then allow us to become mindful of important concepts such as speciation, shared characters and evolutionary novelties, which in turn inform our understanding of phylogenetic patterns and common ancestry. Specific adaptations such as mouth position between benthic and limnetic fish, leaf shape and texture depending on climate are also a great way to introduce adaptation and natural selection.

3. This leads us to the third step of the framework: discussion of the evolutionary processes involved in shaping the patterns felt by the participants.

In sum, any material gathered is capable of being used in a meaningful way, whether a classic textbook example or commonly found specimens. The activity is thus completely adaptable to available material and space, since all phylogenies can be simplified to have more or less branches, and activities can be designed for the participants to spend more or less time in contact with each branch (Fig. 1).

### Multisensory tree of life toolbox

We provide a list of all materials used to represent taxa across the 20 branches (Additional file 2: Table S1) specifying which ones belonged to pedagogic collections from research institutes and education institutions such as museums or aquaria. Photographs of all branches and material display are also available (Additional file 1: Figure S1). All printable 3D models developed from scratch, by scanning real specimens, are available in their final form at MorphoSource.

The tree topology was based on reference phylogenies comprising the taxa of interest (Field et al. 2014; Hedges et al. 2015; Dos Reis et al. 2015) and on the interactive phylogeny *OneZoom* Tree of Life Explorer (Rosindell et al. n. d).

At each branch a volunteer educator provided information to the participants while assisting them in the exploration of the branch-specific materials. Prior to the activity, the educators were provided scripts containing information on what the branch-specific material illustrates, how to guide the people with visual disability to touch the materials and, for the branches where data collection was conducted, the branch-specific activity questions (see Additional file 4: Branch exercises). The scripted branch-specific questions that were given to participants for data collection on the predictability of evolution and basic evolution concepts–like adaptation and natural selection are provided (see methods sections below; Additional file 4: Branch exercises).

To allow for a general perception of the room display and guide the participants with visual impairment independently through the exhibition room, we designed individual haptic hand-maps. These consisted of a blueprint of the room drawn in hot glue on a thin wood plate with 3D information on the phylogenetic path on the floor together with the blueprint of the table display. While this resource was not useful in our implementation, since all participants with visual impairment preferred to be guided trough the activity by a staff member or by their accompanying sighted person; we think that in other contexts, such as museum exhibitions, this might be an inclusive resource that allows the visitor to independently explore the space.

Aspects important to take into account when building a multisensory phylogeny are the fact that partial specimens (like teeth or fur) should always be accompanied by a full model of the organism to make sure that people with visual disability can locate the specific material and make sense of it. Such models, like detailed toy animals, provide a general sense of scale that can help perceive certain biodiversity patterns–e.g. a lynx is bigger than a house cat, as are its teeth, skull and footprints despite the shapes of those structures being extremely similar.

**Fig. 2:**
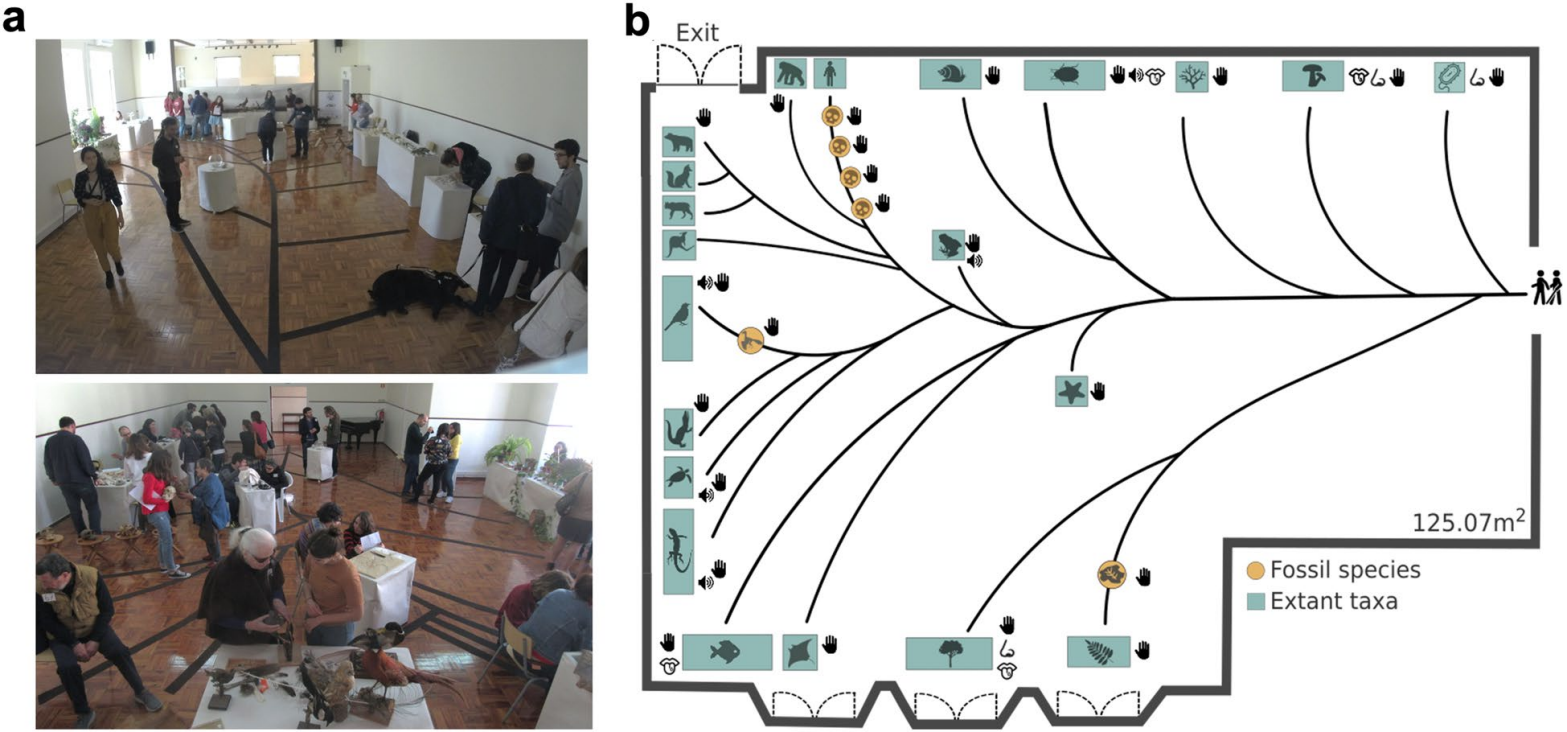
The multisensory tree-of-life (**a**) Photos of the room with the assembled MSToL and (**b**) blueprint of the room with all represented taxa and stimulated senses. The branches of the phylogeny were cut out in carpet making the phylogenetic relationships among groups of taxa accessible for visually impaired people (see Additional file 1: Table S1 for a complete list of materials and Additional file 2: Fig. S1 for detailed branch photos)

Logistics of the room are also very important. In our case, there was not enough space for echinoderms and amphibians to reach the periphery of the phylogeny. These two branches were thus shorter (Fig. 2) which is not optimal as it might inadvertently convey that these taxa are not extant species or that they are somehow ‘less evolved’ than those at the other branch tips. Another aspect to take into account is that, by placing mammals and especially hominids towards the exit and at the top of the room, we might involuntarily contribute to the wrong notion that evolution is a linear process towards humanization so often reinforced by images. However, it’s worth noticing that, in our case, different participants followed completely different routes along the phylogeny, as the movement was mainly dictated by the available educators at the time of branch visit change.

Prior to the MSToL (multisensory tree of life) activity, the volunteer educators received information from the in-house psychologist at the Portuguese Association for the Teaching of the Blind (APEC–Associação Promotora do Ensino dos Cegos) on effective communication with people with visual impairment and the basics of assistance in orienting people with visual impairment.

### Participant data collection

In order to evaluate our activity, data were collected from 25 participants with visual impairment (15 women and 10 men) and 23 sighted participants (17 women and 6 men; Additional file 3: Table S2). All data were collected *in loco* at the Portuguese Association for the Teaching of the Blind (APEC) on the day of the outreach activity (12 of March, 2019), before and after participation, following the questionnaire provided (Additional file 5: Questionnaire).

The questionnaire included personal data, an exercise of true or false and a word association exercise. Both exercises were scored to allow us to assess and compare the participants’ knowledge on basic concepts of evolution, before (basal knowledge) and after the activity.

We recorded, for each participant, demographic data–age, gender–and also their education level, interest in biology/evolution and visual capacity. Education level of the participants was coded according to the Portuguese education system as follows: Primary School (PS) 1st to 4th grades; Middle School (MS) 5th to 9th grades; High School (HS) 10th to 12th grades; bachelor’s degree (B) and master’s degree (M). B and M are referred to as ‘post-secondary education’ throughout the manuscript. The level of visual impairment of the participants was assessed by asking each individual which one of the following 4 levels they identified with: ‘*No disability*’, which includes people with glasses correcting for standard vision levels; ‘*Moderate vision loss’*, which refers to people with low vision with perception of shapes and colors (includes people with corrective lenses); ‘*Deep vision loss with residual light perception*’, which refers to people with extremely low vision but that can still perceive some light variation; and ‘*Profound vision loss without any light perception*’, which refers to people can not receive any visual cues.

Throughout the manuscript, when we refer to ‘people/participants with visual impairment’ we are referring to the whole spectrum of visual impairment, specifically defined above for our panel of participants. When we refer to ‘people/participants who are blind’ we refer to people who identify with the fourth group described–profound vision loss without any visual cues. We additionally recorded how long the person has lived with visual impairment.

Data were also collected, through an online questionnaire, from 15 out of 24 volunteer educators to assess their emotional response to the activity and understand if the inclusive activity was mutually beneficial: for participants and educators.

The resulting data allowed us to (1) generally evaluate the effectiveness of the designed haptic activities, (2) establish baseline data about evolution knowledge and interest within a subset of the Portuguese community with visual impairment, (3) determine whether participants enjoyed the activity, and (4) begin to determine how such activities might improve the learning of evolution.

Data collection was approved by the University of Bath, Department of Psychology, Research Ethics Committee (code 17–273). All participants provided informed consent prior to participating, participated voluntarily and were informed of their right to withdraw participation at any point during data collection.

All data collected is presented anonymously in Additional file 3: Table S2.

### Branch-specific exercises

In nine of the 21 branches, participants were asked branch-specific questions (Additional file 4: Branch exercises). These questions related to the haptic materials available on the table and were primarily exercises of prediction of phenotypic change, or adaptation scenarios about environmental change. In total, there were 29 questions focused on organisms’ evolutionary responses to certain environmental changes, designed to assess the participants’ understanding of adaptation, fitness, environment-phenotype associations, gradualism and natural selection, and their predictive ability of evolutionary change. Participants were not given possible answer options. They were asked the scripted questions (Additional file 4: Branch exercises) and the volunteer educator scored their answers in the record sheet according to the level of evolutionary thought: Answers in the ‘maybe’/‘I don’t know’ category were scored as 0; if the answer was not the known outcome of the evolutionary process but involved plausible evolutionary outcomes (extinction, mutation) it was scored as 1; if the answer took into account natural selection and adaptation it was scored as 2, with one extra point for the four questions in which gradualism or selection strength was considered. Finally, if the answer was in the ‘nothing changes’ category and did not consider any evolution outcome, it was scored as − 1.

Since not all participants answered all branch-specific questions, the standardized branch-score was calculated for 24 participants with visual impairment and 17 sighted participants as the sum of individual answer scores divided by the number of questions answered by the participant.

The ‘prediction score’ regards the subset of 18 questions, spanning 6 branches (three questions on the plant branch, three on corals, four on bony fish, three on molluscs, one on hominids and four on turtles) that require the participant to predict the outcome of an environmental change and was calculated and standardized as described above, for 23 participants with visual impairment and 17 sighted participants.

### Word association exercise

We wanted to know if people were familiar with the scientific terms necessary to understand the basics of the theory of evolution and to what extent terms that are essential or might promote its misunderstanding were commonly associated with the concept of ‘evolution’. For this we designed a word association exercise where participants were read a list of 33 words, one at a time, and upon hearing each one reported whether a given word was instinctively associated with evolution by responding ‘True’ or ‘False’ (Refer to Fig. 4 for the complete list of words and Additional file 5: Questionnaire). Words were scored as – 1 if the word is usually associated with misunderstanding of the evolutionary process (e.g. perfecting); as 0 if the word is neutral and unnecessary to explain the theory of evolution (e.g. science); as 1 if the word is not necessary to explain the theory of evolution but it is related to it (e.g. Darwin) and as 2 if a word is necessary and fundamental to explain and understand evolution (e.g. natural selection). Individual scores were calculated by summing the word scores of a participant. Because we wanted to evaluate instinctive answers, a participant had on average 3 s to provide an answer and if hesitation was long, it was recorded as ‘non-association’. When participants declared that they didn’t know it was recorded as ‘not applicable–NA’.

We recognize that the experimental design of this exercise has flaws: ideally, the number of incorrect terms should be similar to the one of correct and essential ones and the order in which the words were presented to each participant should have been randomized (all participants were read the same words in the same order). However, we believe that the report of the results might inform future outreach activities and thus present it as basal data and not as a proof of principle.

### Data analysis

All data analyzed for comparisons (mean differences) and correlations were checked for deviations from assumptions using a Shapiro test (α = 0.05). If data distribution did not significantly differ from normal distribution, parametric versions of the relevant statistics were used, otherwise non-parametric statistics were used (Student’s *t*-test Vs. Mann–Whitney U test for mean differences; and Parametric (bivariate) Vs. Non-parametric (Spearman) for correlations). Each result is accompanied by the specific test performed in the results section. Data were always standardized for the specific number of participants comprising the dataset.

We emphasize that our sample sizes are small which translates into data limited in its power and thus we provide these as explorative baseline data regarding our specific activity, which might inform future research questions on inclusive pedagogy.

All analysis and data visualization were performed in R version 3.6.0 (R Core Team 2019) and all raw data and analysis code are available to allow full analysis replication (Additional files 3 and 8, respectively).

A description of all figures and images presented below, accessible for people with visual impairment, is presented in Additional file 7.

## Results

### The multisensory tree of life for all

The multisensory Tree of Life (referred to as ‘MSToL’ from here on) occupied 125 square meters and was composed of branches representing 21 extant taxa–plus eight fossil species. The majority of materials consisted of real biological samples (Fig. 2; Additional file 1: Fig. S1 for detailed photos per branch; Additional file 2: Table S1 for a list of materials, source, sense stimulated and evolution concepts explored, per branch). All five basic senses–hearing, smelling, touching, tasting, seeing–were stimulated across the phylogeny, with touch being stimulated by all displayed material, becoming the main source of information acquisition for people with visual impairment.

Each consenting participant–23 sighted and 25 with visual impairment–contributed to data collection through a questionnaire applied before and after experiencing the MSToL room, and branch-specific quizzes. The resulting data allowed us to generally evaluate the success of the activity in terms of learning, and to establish baseline data on evolution knowledge for a subset of the Portuguese community with visual impairment.

### Implementation: the hardest and easiest taxa to represent

Upon consulting with blind members of the community of the Portuguese association for the teaching of the blind (Associação Promotora do Ensino dos Cegos, APEC), it became clear that touch would be the most inclusive sense to explore the phylogeny. Thus, for the first step—allowing participants with visual impairment to assess biodiversity accurately—haptic communication was essential. Because we wanted all participants to experience real biodiversity patterns, we mainly acquired real specimens and biological samples that could be touched. This can, however, raise challenges for the representation of some taxa: museum collections with scientific value are usually unique and fragile, which hinders their free manipulation; live specimens pose animal welfare concerns; and commercially available models can be inaccurate and lack the detail needed to fully comprehend the range of biodiversity patterns. Several of these difficulties applied to the arthropods, which proved to be the most challenging taxa to translate into multisensory communication. We addressed this difficulty by incorporating 3D-prints of in-house μ-CT scanned specimens, insect sounds, edible insects, exuviae and structures built by arthropods, such as hives (Additional file 1: Fig. S1 e to g). Collaboration with education centers with available pedagogic collections was imperative to obtain material that accurately depicts biodiversity (Additional file 1: Fig. S1; Additional file 2: Table S1). The most effective branches in terms of degree of ‘effort to find material’ with respect to the magnitude of ‘activities and information that can be extracted from it’ were plants, birds and primates. A great variety of plants are commercially available and they are ideal to develop activities focused on plant-pollinator coevolution and phenotype-climate adaptations (Additional file 1: Fig. S1 t to w). For birds, songs, calls and full specimens of game species are easily obtainable and optimal for activities focused on phenotype-environment adaptations (Additional file 1: Fig. S1 n). The evolution of humans and related primates holds, generally, particular interest for the public (Pobiner 2012). Anthropological model collections can be expensive but are usually available at universities where anthropology is taught, which can be invited to lend this resource. A collection of hominid skulls is a great resource for discussion of human evolution and for understanding common ancestry while dismantling the myth that “*Homo sapiens* descends from monkeys”.

While teeth and different types of fur can be very interesting resources to discuss adaptation, without complete spatial or morphological information they can be confusing for participants with visual impairment, especially for those born blind. To avoid this we made sure that models of the full organism were available for any type of partial specimen, which the participants reported to be extremely useful.

### Public attendance

During the 12 h of activity, we received an estimated total of 100 participants, 60 of which had visual impairment. We did not restrict the amount of time to explore the MSToL, allowing the participants to do so at their own pace.

Participants, especially those with visual impairment, tended to remain in the room more than the predicted one hour, with some remaining for as long as four hours. This should thus be a full day activity at minimum, and ideally a multiple-day activity.

A subset of consenting participants responded to a standardized questionnaire which included general, as well as branch-specific, questions (Additional file 5: questionnaire), administered both before and after experiencing the MSToL room. The sample comprised 25 adults with visual impairment, with average age 62 (range: 18 to 82) and 23 sighted adults, with average age 58 (range: 24 to 90).

The first assessment of the data showed that the majority of participants with visual impairment had profound vision loss without perception of any visual cue, referred to as blindness throughout the manuscript (13/25) and only a minority (4/25) had moderate vision loss, with perception of shapes and some colors, and eight out of 25 had deep sight loss with residual light perception (see methods for details of the participants’ self-assessment). The majority of participants with visual impairment had the impairment for more than half of their lifetime, with only seven living with it for less than that period.

### Preferences: touch and birds

The sense preferences for experiencing the MSToL were consistent across participants, regardless of visual ability. Touch was listed as the most informative sense by 83.3% of participants with visual impairment and 50% of sighted participants (Additional file 3: Table S2). On the other end, gustation was listed as the least informative sense by 93.7% of participants with visual impairment and 53.3% of sighted participants (Additional file 3: Table S2), which was also the least stimulated sense across the phylogeny (Fig. 2b, Additional file 1: Table S1).

Despite an overall scattered preference across taxa, people with visual impairment showed slight predilection for the bird branch: five out of 19 people with visual impairment chose the birds as their favorite branch (Additional file 3: Table S2). This tendency might have been influenced by the communication skills of the educator responsible for the birds’ branch, but it is worth noting that even for pragmatic reasons birds are a good taxonomic group to invest in further, more focused, activities. This taxon allows multisensory activities on adaptation and evolution to be design relatively easily due to the ease of acquiring diverse feather types, the abundant availability of taxidermy specimens with different bill shapes among game species, and also the widespread availability of bird song recordings, easily adding an auditory component.

### Despite lack of access to education, participants with visual impairment are interested in, and understand, evolution

**Fig. 3:**
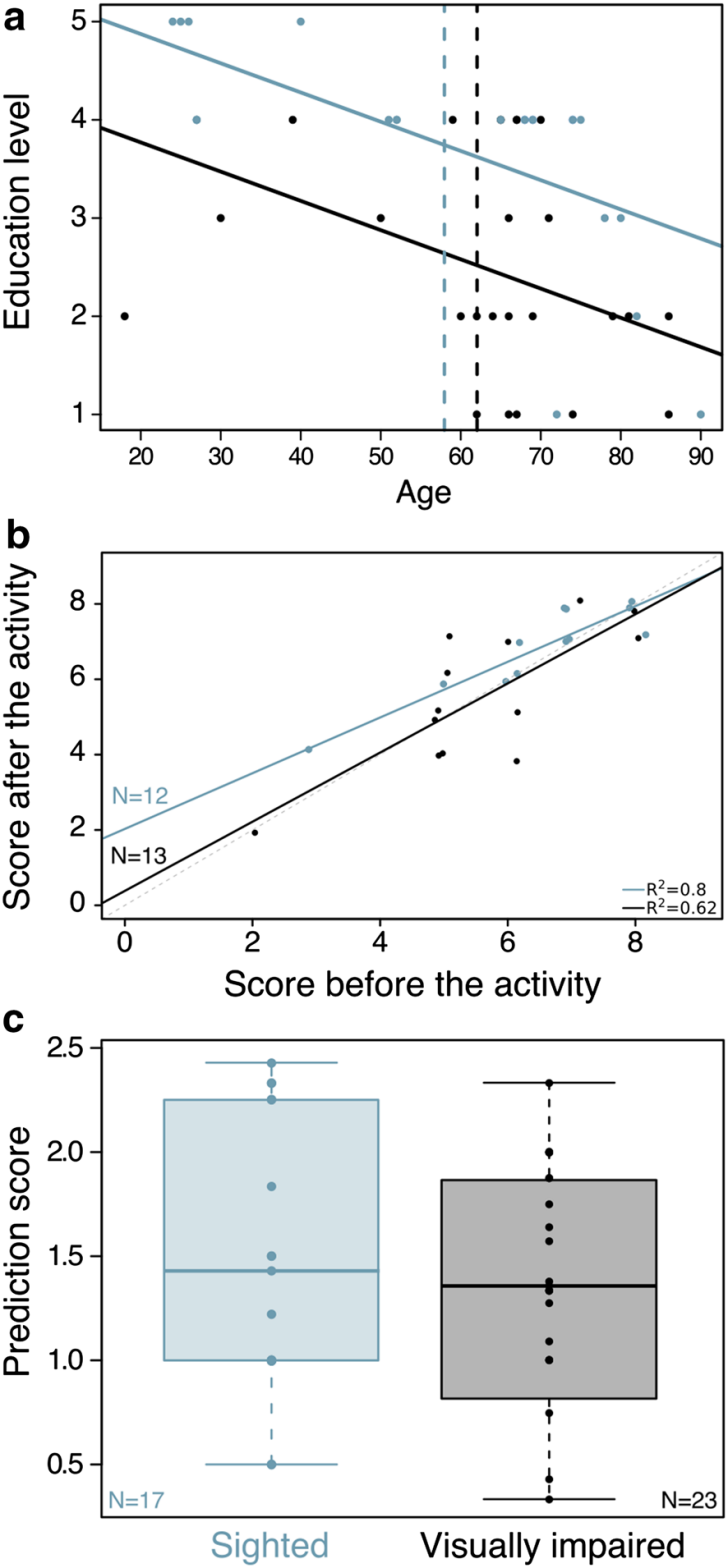
Participant education data and scores, based on basic knowledge of evolution before and after the activity, and prediction of evolution outcomes. Blue data refer to sighted participants and black data to participants with visual impairment. **a** Depicts the relationship between participants age and education level. Lines visualize the linear model per participant category and vertical dashed lines mark the average age of each group. **b** Refer to the scores of true or false questions on basic evolution concepts applied before and after the MSToL activities. Lines show the correlation between before and after score. For sighted participants (blue) and visually impaired participants (black), R squared values are depicted from a regression analysis with ‘Score after the activity’ dependent on ‘Score before the activity’. **c** Visualizes participants’ predictive ability in scenarios where environments shape phenotypic responses, following the same color code

We found that participants with visual impairment had a lower level of educational attainment (Fig. 3a). The majority of participants with visual impairment had not enrolled in high-school education (56%, 14 out of 25), only seven participants with visual impairment had attained post-secondary education, and none held a master’s degree. In comparison, the majority of sighted participants had attained bachelor’s degrees (59.1%, 13 out of 22; Fig. 3a). Only two of the seven participants with visual impairment (28.6%) with post-secondary education had taken courses in biology and evolution (at the bachelor’s degree level), compared to six of the 17 sighted participants (35.3%; Table S2).

Interestingly, when enquired regarding their interest for evolution (for questionnaire scale description refer to Additional file 5: questionnaire; and for individual data refer to Additional file 3: Table S2), a similar percentage of participants from both groups described themselves as having a lot of interest in evolution (28% with visual impairment; 39% sighted). Indeed, two participants with visual impairment shared with the educators that they wanted to become biologists, having given up because “it was too visual”, making it clear that the biological sciences are not equally accessible to everyone. However, basal knowledge, which was measured by the amount of correct answers scored before the activity, was quite high for both groups: all participants tended to score high both in the true and false questions and in the word association exercise (Additional file 6: Fig. S3). We did not find statistical differences in average performance between sighted people and people with visual impairment (Fig. 3; Additional file 6: Fig. S2 and S4), based on learning (Fig. 3a; Pearson correlation: r = 0.79 for participants with visual impairment; Spearman correlation rho = 0.81 for sighted) and prediction scores (Fig. 3b; *t*-test: *p*-value = 0.77). However, the higher ‘learning’ and ‘prediction’ scores tended to belong to sighted participants, while the lowest scores tended to be recorded for participants with visual impairment (Fig. 3; Additional file 6: Fig. S2 and S4; refer to Methods for a detailed description of all score metrics). Based on the results from the branch-specific activities, it is also notable that, when enquired about the consequences of environmental changes, both sighted people and people with visual impairment could successfully predict evolution outcomes in terms of expected phenotypic changes (Fig. 3b; refer to Additional file 4 for detailed description of prediction exercises), which shows a general basic understanding of the mechanisms of natural selection and adaptation.

The fact that we have a small sample size, together with the substantially high basal knowledge (Additional file 6: Fig. S3) found for both participant groups (Fig. 3a), makes it difficult to assess the true effectiveness of the activity based exclusively on *in loco* data.

An inclusive description of all figures and plots above-mentioned is presented in the Additional file 7.

### ‘Common ancestor’ and ‘Natural Selection’ become more familiar terms, but artificial selection and neutral evolution might be hard to grasp without specific activities

**Fig. 4:**
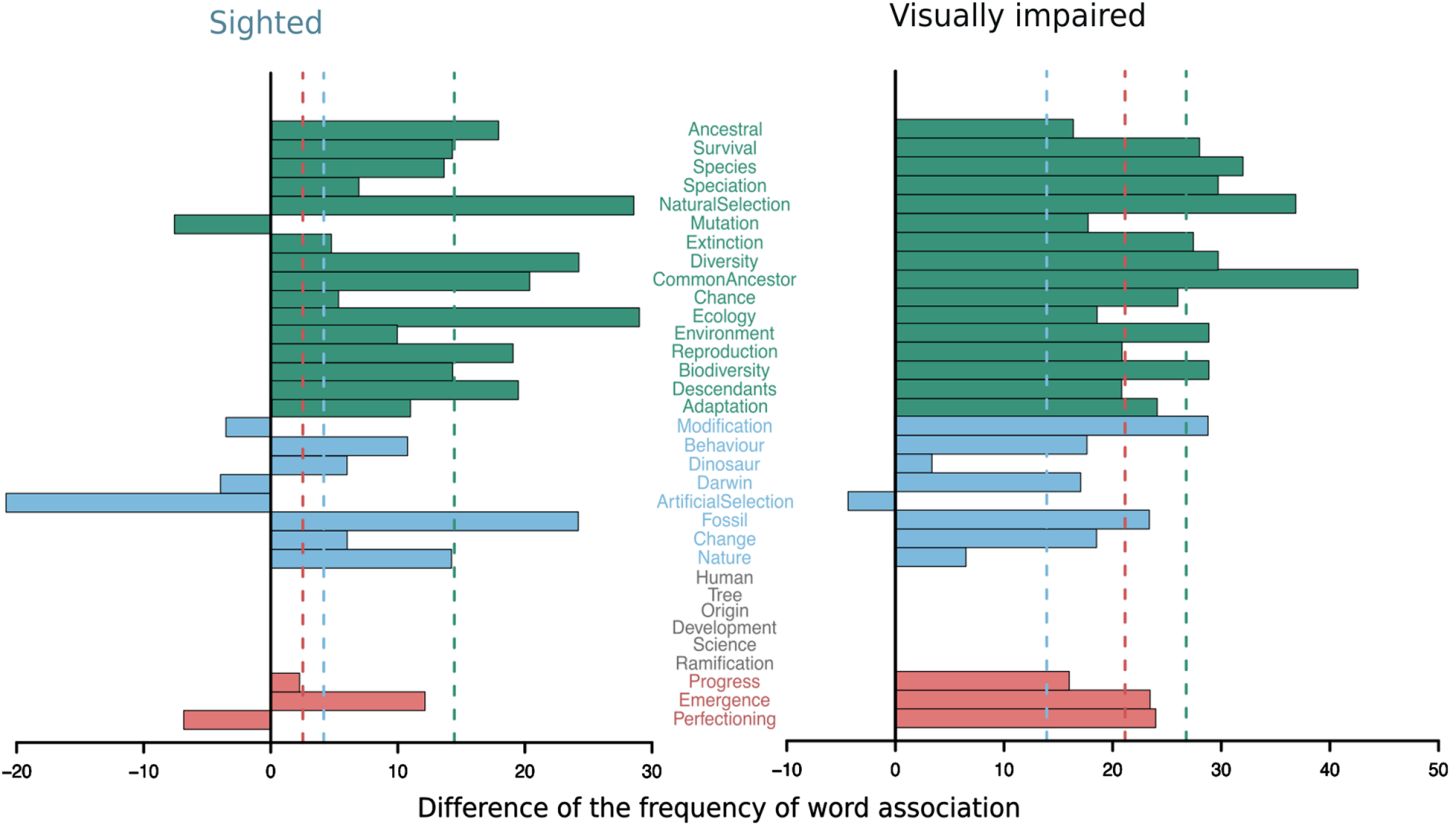
Terms associated with the concept of evolution before and after the MSToL activity. Metric shown is the difference of percentage of people associating the word with evolution after the activity minus the percentage of people associating the word with evolution from the beginning. In dark green are terms essential to understand evolution, in blue are words related, but not essential; in grey neutral terms, and in red terms that can be misused or promote the misunderstanding of evolution. Notice that neutral term bars are absent because they were equally associated before and after the activity (see Additional file 6: Fig. S5). Vertical dashed lines depict the average difference of each word group, following the same color scheme

At the beginning and end of the activity, participants were read the same list of 33 terms and asked if they instinctively associated them with the concept of ‘evolution’ (Fig. 4; refer to the methods section for details). This meant to assess whether terms that are essential to understand evolution–like adaptation and common ancestor–were clearly present in the participant’s minds.

After the activity, association of terms increased in general. Overall, the terms that increased the most were ‘Common ancestor’ and ‘Natural selection’ for the participants with visual impairment; and ‘Ecology’ and ‘Natural selection’ for sighted participants (Fig. 4, Additional file 6: Fig. S5). All these terms were heavily used on the scripts provided to the educators. However, for the participants with visual impairment, the association of terms that can lead to misunderstanding evolution–such as perfecting and progress–increased more (Fig. 4, Additional file 6: Fig. S5).

All participants associated all neutral terms–such as tree or ramification—with ‘evolution’ both before and after the activity (Additional file 6: Fig. S5).

Interestingly, the term “artificial selection” decreased in association for both groups. This is not surprising since there were no artificial selection activities or mentions. This term was not used during the workshop, as the majority of the examples and predictive exercises were based on well-studied responses to natural selection, such as the stickleback fish plating reduction or the food-availability-driven beak morphology of birds.

Also interesting is that while ‘mutation’ was highly associated with evolution by both groups, already before the activity (84% of sighted and 86% of participants with visual impairment), ‘chance’ was not. Together with the fact that ‘Progress’ and ‘Perfecting’ tended to increase, this might signal that a linear view of evolution towards perfection and humanization is rooted in the participant minds and calls for the need of including neutral forces in outreach activities to avoid promoting wrong or extremely adaptationist views of evolution.

### Volunteer educators’ emotional experience reveals no discomfort in communicating with participants with visual impairment and that through teaching they also learn a lot, while having a lot of fun.

To assess the benefits of inclusive outreach activities for the educators, we conducted a brief post-activity questionnaire on the volunteer educators.

**Fig. 5:**
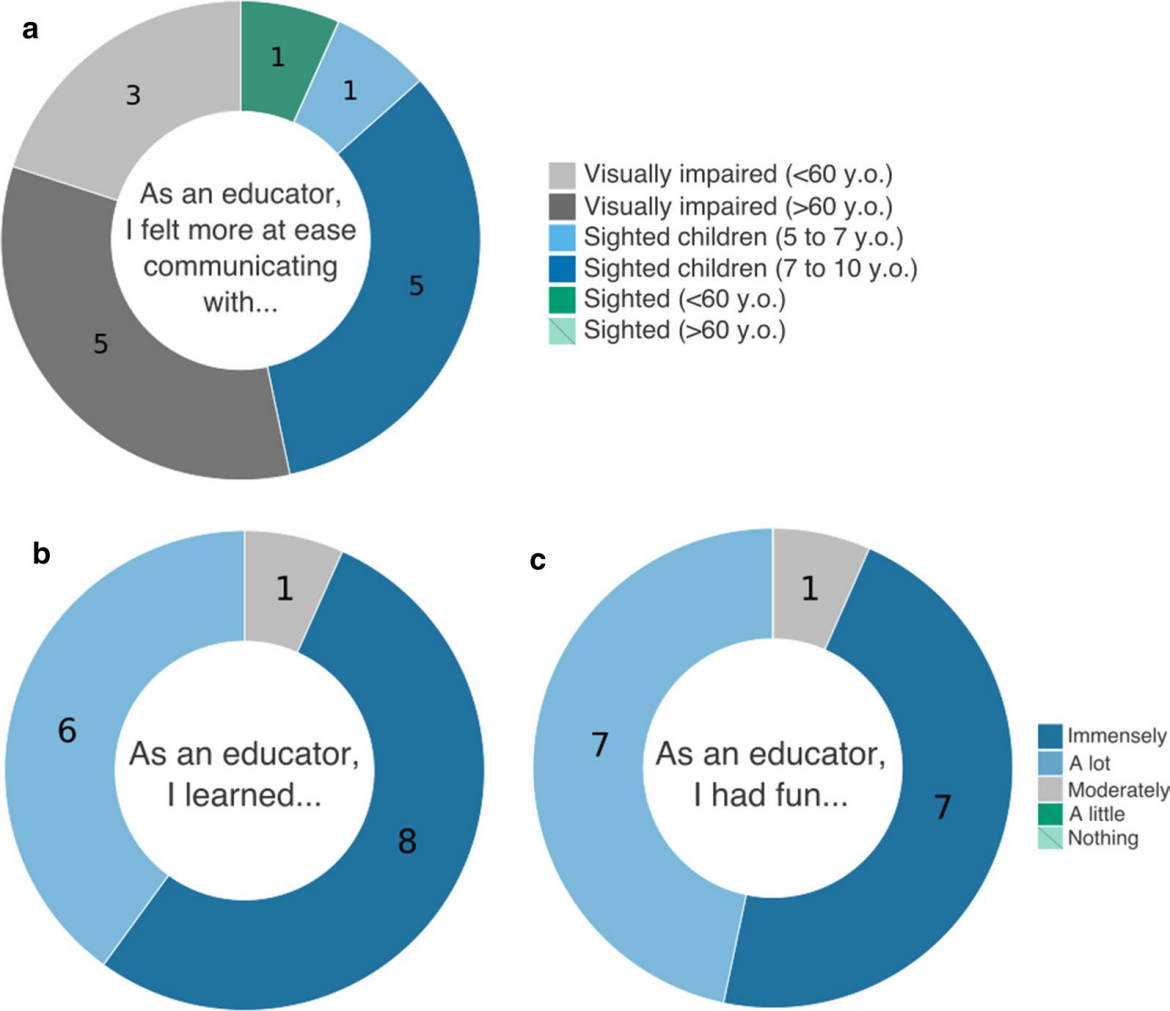
Volunteer educator emotional experience. Visualization of data collected from 15 out of 24 volunteers on **a** the public with which the communicators felt more at ease; **b** the feeling of having learned while educating and **c** the feeling of having fun while educating the public. Numbers within circle are the recorded answers for each option

Despite the majority of volunteers (93.3%; 14/15) not having previous experience communicating with people with visual impairment, during the activity, 53.3% felt more at ease communicating with people with visual impairment, especially with those participants above 60 years old (Fig. 5a). When asked what was their favorite aspect of communicating with participants with visual impairment, the volunteers expressed feelings of empathy and mentioned discovering the world from a new perspective. Furthermore, the great majority of volunteer educators reported that they learned immensely (Fig. 5b) while educating, especially communication skills, and reported they had a lot of fun while teaching evolution (Fig. 5c).

## Discussion

The development of the multisensory tree-of-life and the data collected during the activity provide evidence for three main arguments: (1) there is a clear lack of accessibility to evolutionary biology education for people with visual disability, despite their interest in the matter; (2) the classic examples of evolution are capable of being transformed into multisensory activities; (3) touch and haptic models are essential for people with visual disability and a plus for sighted participants, making haptic activities the most powerful resource to increase accessibility and inclusion, benefiting everyone, independently of physical impairment.

### Lessons learned to provide a comfortable and stimulating experience for people with visual impairment

As a pioneer activity, we were faced with a lot of hurdles due to inexperience. With this publication we hope to reduce those for anyone reproducing this activity or creating similar ones. However, our main and most valuable lesson concerns communication. As sighted organizers we needed to make sure that our ideas and translation of visual into haptic were indeed accessible to people with visual impairment; and that the MSToL room was comfortable for everyone. Consulting with people with visual impairment or blindness is absolutely indispensable at every step. Initially, we had designed the activity as having braille instructions at the tables, which greatly diminishes the amount of volunteer educators needed. However, our blind consultant informed us that in our local community of people of visual impairment, only a small minority knew how to read braille. Another very important insight was the fact that people who have lived with visual impairment for different lengths of their lives will have different sensitivities to more subtle textures, might be more or less comfortable with the volume of sound in the room, and might be more or less experienced in navigating a room with floor textures. Thus, tripping hazards should be avoided when planning the floor phylogeny texture and the disposition of the branches—flat carpet for the phylogeny and tables closer to the walls of the room were our optimal design. If there are participants with motor disabilities or on wheelchairs, the height and shape of the display tables also need to be considered for accessibility. A big part of offering a safe and stimulating environment for people with visual impairment is the acoustics of the room: a lot of echo and noise easily becomes overwhelming. To avoid this, it is important to control the flow of people in the room and the volume of the sounds within. Following the available guidelines together with communicating and consulting with the local community of people with visual disability are crucial to ensure a comfortable learning experience for everyone.

### Be careful with the ‘evolution ladder towards humanization’

The aspect that we believe is the most urgent to improve in this and other activities, is the fact that the public easily retains the misconception of evolution as a linear processes towards humanization. Despite directly countering this idea through the branching phylogeny patterns presented, a question that seemed to hold low scores, across all participants, both before and after the activity, asks if ‘mammals are more evolved than fish’ (Refer to Additional file 6: Fig. S3 for individual question scores). In future activities, attention should be paid to the fact that having mammals and, more specifically, the *Homo* branch ‘higher’ on the phylogeny display–in our case towards the end of the experience for someone who started at the root (Fig. 2)–might promote the incorrect, and quite common, notion of evolution ‘progressing’ linearly towards humanization and taxa that are evolutionarily closer to our species.

Thus, as something to improve, topology display (*e.g.* display mammals more to the side instead of at the top, do not make *Homo* the last visited branch) and language use (*e.g.* never use ‘more primitive’ or ‘basal’ for any extant taxon) should be mindful of inadvertent contribution towards the adaptationist and directional evolution narratives.

### Towards more effective and inclusive outreach activities

Global estimates are clear regarding the under-representation of people with disability in STEM, both in the classroom and in the academic community (Moon et al. 2012). Under this *status quo*, people with visual impairment are denied access to knowledge and to participation in scientific communities. Consequently, we lose diversity of thought and experiences that could promote more stimulating ways of teaching that everyone could benefit from.

In evolutionary biology, besides displaying data and concepts, images are a source of interest and wonder for biodiversity, which fuels curiosity. Being so, the promotion of scientific literacy demands the translation of evolution’s patterns to senses other than vision. Although text and audio descriptions of graphical representations are useful, students with visual disability have reported that many important details are left out or misinterpreted by the translator (Shute et al. 2005). Furthermore, as the complexity of visual content intensifies so does the challenge of presenting it through auditory cues (Shute et al. 2005).

In a comparative study on sighted science students, tactile learners retained and understood concepts better, while also enjoying their lessons much more (Pashler et al. 2009). The preference of both MSToL participant groups (with and without visual impairment) for ‘touch’ as a learning sense, and the overall positive global learning scores reported in our activity, further suggest ‘touch’ as a generally inclusive and powerful vehicle of information delivery.

In fact, evolution concepts can be ideal for tactile learning, because much of the visual content represents descriptions of morphological and environmental variation, easily translated into 3D haptic images. When these resources are incorporated into science teaching, interest can increase for both sighted and blind students (Hasper et al. 2015). Thus, as shown by growing evidence from life sciences (Fraser and Maguvhe 2008), communication should be multisensory to increase teaching effectiveness for all students.

## Conclusion

Without inclusive approaches, students with visual impairment often lose motivation due to real or perceived physical barriers to knowledge acquisition (Bell and Silverman 2018). However, when knowledge is made accessible they can realize their potential just as sighted students do (Sahin and Yorek 2009). Therefore, the inclusion of multisensory activities in outreach, which we have shown to be quite accessible for a lot of branches of the tree-of-life, can have important academic and social impacts. Just like museums, outreach activities should be ‘inclusive and polyphonic spaces that address present social challenges and promote active partnerships with and for diverse communities, contributing to human dignity and social justice, global equality and planetary wellbeing’ (Sandahl 2018). Involving complementary senses on future activities will not only promote equity for those with disability, but also move us faster towards an inclusive and diverse scientific community, and towards a public more aware of biodiversity, evolution and our connection to it.

## Supplementary Information

**Additional file 1.** List of all the materials used in the MSToL per branch, their source and respective evolution concepts explored.

**Additional file 2.** Detailed photos of all branches in the MSToL.

**Additional file 3.** Anonymised raw data.

**Additional file 4.** Description of the branch exercises applied to assess prediction ability.

**Additional file 5.**  Questionnaire for data collection applied to participants before and after the MSToL experience.

**Additional file 6.** Additional figures and data visualization.

**Additional file 7.** Inclusive description of the publication figures for people with visual disabilities.

**Additional file 8.** R code for data analysis.

## Data Availability

All raw data (anonymous version) and code used for analysis are available (Additional files 3 and 8, respectively). μCT scan images and 3D meshes that allow reproduction of the 3D printed material is available on MorphoSource (project title is same as publication); All materials used are described in detail, per branch, in Additional file 1. An inclusive description of the data shown is presented in Additional file 7.
