# Supplementary material for: evALLution: making basic evolution concepts accessible to people with visual impairment through a multisensory tree of life
Source: Evolution (N Y). 2021 Mar 11;14(1):5. doi: 10.1186/s12052-021-00143-1 (PMC7952356; doi:10.1186/s12052-021-00143-1)
Supplement: Supplementary file 1 — Additional file 1. List of all the materials used in the MSToL per branch, their source and respective evolution concepts explored. [file 12052_2021_143_MOESM1_ESM.pdf]

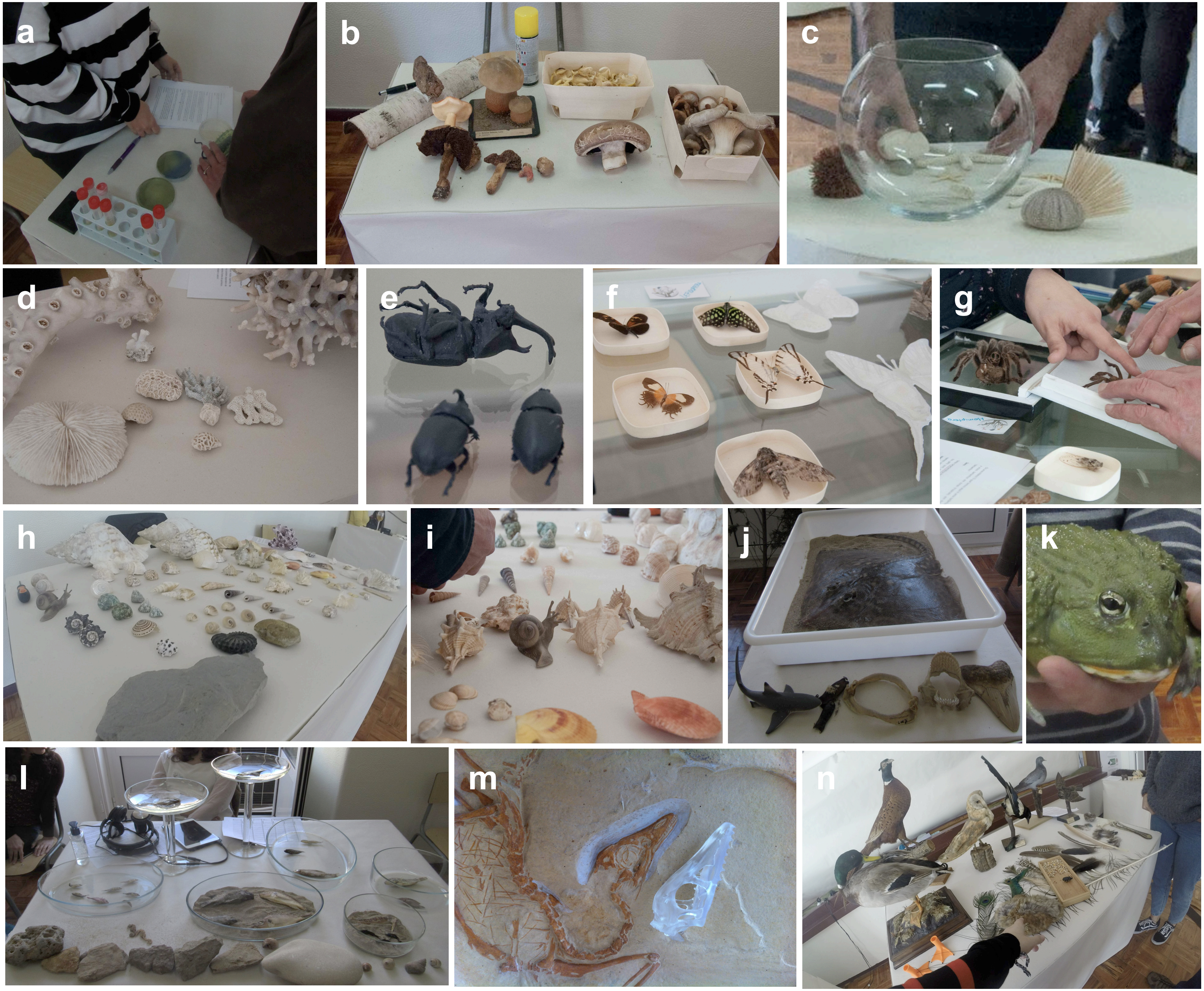
a
b
c
d
e
f
g
h
i
j
k
l
m
n

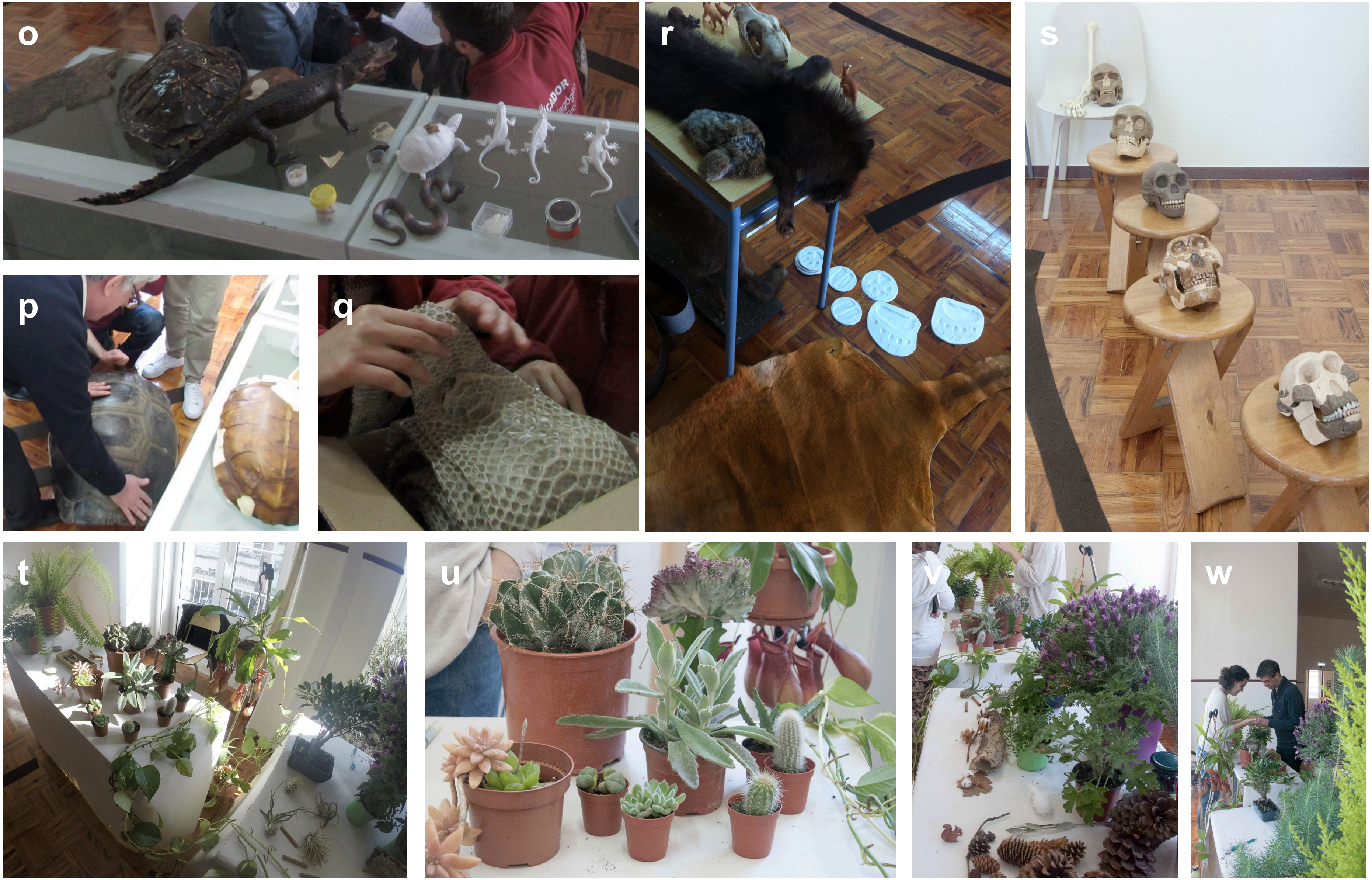

Figure S1. Photographs of the material representing the 20 taxa comprised in the multisensory phylogeny.
Photographs by Rita Justino, Marisa Xavier and Telma G. Laurentino
