# Supplementary material for: evALLution: making basic evolution concepts accessible to people with visual impairment through a multisensory tree of life
Source: Evolution (N Y). 2021 Mar 11;14(1):5. doi: 10.1186/s12052-021-00143-1 (PMC7952356; doi:10.1186/s12052-021-00143-1)
Supplement: Supplementary file 2 — Additional file 2. Detailed photos of all branches in the MSToL. [file 12052_2021_143_MOESM2_ESM.pdf]

Table S1. List of materials used in the multisensory phylogeny
Asterisks mark material borrowed from pedagogic collections of education centers (Lisbon Lisbon's Zoo, Lisbon Oceanarium, Zoological Institute from the University of Basel, or Sciences Faculty from the University of Lisbon). For photographs of the referred material see Fig. S1.
♯ For questions for data collection, see sup. material 'Scripted branch-specific exercises'.

Branch	Materials	Sense stimulated	Data collection in this branch? (Yes/No)♯	Concepts explored
Bacteria	*E. coli* model by Ein-O Science	Touch	N	Biodiversity Adaptation (antibiotic resistance)
	Filtered E. coli medium in falcon	Olfaction		
	Petri dish for scale	Touch		
Fungi	4 species of edible mushrooms	Touch; Taste	N	Biodiversity
	Silly string (mycelium mimic)	Touch		
	Birch bark with shelf mushroom	Touch		
	4 species of dried mushrooms	Touch		
	2 models of mushrooms	Touch		
Echinoderms	Koosh ball (ambulacracria feet mimic)	Touch	N	Biodiversity<br><br>Extinction
	Sea urchin skeletons from 3 species (one with glued toothpicks)	Touch		
	Sand dollar fossil	Touch		
	Starfish skeleton from 2 species	Touch		
Corals	Ceramic replica*	Touch	Y	Adaptation
	9 samples of different colony morphology	Touch		
Arthropods	3 Tarantula exuviae	Touch	Y	Biodiversity<br><br>Adaptation (to different environments and predator pressures)<br><br>Character displacement (songs)<br><br>Sexual selection (beetle horns)
	4 specimens of butterfly species	Touch		
	1 specimen of moth	Touch		
	2 haptic models of butterfly (pattern recreated with hot glue in fabric)	Touch		
	1 specimen of cicada	Touch		
	5 cicada larvae exuviae	Touch		
	Cicada sounds	Hearing		
	3 3D printed beetles	Touch		
	Edible beetle larvae	Touch;		

		Taste		
	Beetle sounds	Hearing		
	Edible grasshoppers	Touch; Taste		
	Grasshopper sounds	Hearing		
	Edible crickets	Touch; Taste		
	Cricket sounds	Hearing		
	5 bumblebee specimens (2 species)	Touch		
	2 bee specimens	Touch		
	Part of a paper wasp's nest	Touch		
	Honey	Taste		
Molluscs	Haliotis shells (several sizes)	Touch	Y	Biodiversity<br><br>Adaptation (to predator pressures)<br><br>Reproductive fitness
	Bilvalvia shells of 14 species	Touch		
	Gastropoda shells of 21 species	Touch		
	2 fresh sea snails (fish market)	Touch		
	1 model of snail by Schleich	Touch		
	Cylindrical rasp tool (mimic radula)	Touch		
	3 ammonite fossils*	Touch		
	Cuttlefish skeleton (fish market)	Touch		
Cartilaginous fish	Megalodon fossil tooth*	Touch	N	Biodiversity<br><br>Extinction
	Shark model by Science4You	Touch		
	Shark Jaw*	Touch		
	Shark eggs	Touch		
	Stingray jaw*	Touch		
	Stingray specimen (fish market)	Touch		
Amphibians	Live specimen of packman frog (with caretaker)	Touch	N	Biodiversity<br><br>Adaptation (to terrestrial habitats)
	Amphibian calls from 4 species	Hearing		
	Water jell balls (mimic eggs)	Touch		
Fish	8 specimens of threespined stickleback (4 complete plated morph; 4 low plated morph) *	Touch	Y	Biodiversity<br><br>Adaptation (to depth, food niches and predator pressures)
	2 specimens of sea	Touch		

	horse*			
	2 Tanganyika catfish specimens*	Touch		
	1 Tanganyika eel specimen*	Touch		
	1 Tanganyika killi fish specimen*	Touch		
	1 mormirid*	Touch		
	Mormirid electric sounds	Hearing		
	10 Tanganyika cichlid specimens*	Touch		
	Coral reef fish sounds	Hearing		
Archaeopteryx	Fossil replica	Touch	Y	Extinction
	3D printed skull reconstruction	Touch		
Birds	Mallard duck (taxidermy)	Touch	Y	Biodiversity Adaptation (to food niche)
	Barn owl (taxidermy)	Touch		
	Pheasant (taxidermy)	Touch		
	Magpie (taxidermy)	Touch		
	Pigeon (taxidermy)	Touch		
	Starling (taxidermy)	Touch		
	Spoon-bill skull*	Touch		
	Keel bone	Touch		
	Assortment of different Peacock feathers	Touch		
	2 Nests	Touch		
	Food resources (to match with beak shape): rabbit; insect, seeds	Touch		
	Vocalizations for all represented species	Hearing		
Reptiles	1 crocodile specimen*	Touch	Y	Biodiversity Adaptation (to predator pressures) Extinction
	Turtle 3D model*	Touch		
	Shell of a giant tortoise*	Touch		
	Specimen of marine turtle (taxidermy) *	Touch		
	Turtle egg shells*	Touch		
	Turtle sounds	Hearing		
	Turtle shell scales*	Touch		
	3D printed monitor lizard skull	Touch		
	Models of 3 species of lizards	Touch		
	Models of snake	Touch		
	Snake specimen	Touch		
	Python skin*	Touch		

Marsupials	Kangaroo skin	Touch	N	Biodiversity
	Kangaroo model by Schleich	Touch		
Carnivores	Lynx footprints	Touch	N	Biodiversity<br><br>Adaptation (to food niche)
	Lynx model by Schleich	Touch		
	Cat model by Schleid (scale for lynx)	Touch		
	Bear footprints	Touch		
	Bear model by Schleich	Touch		
	Fox pelt	Touch		
	Fox specimen (taxidermy)	Touch		
	Dog skull*	Touch		
	Vocalizations of all represented carnivores	Hearing		
Primates	Chimp skull model*	Touch	Y	Biodiversity<br><br>Human evolution<br><br>Adaptation (jaws and brain volume)
	Chimp model by Schleich	Touch		
	Australopitecus afarensis skull model*	Touch		
	Paranthropus boisei skull model*	Touch		
	Homo erectus skull model*	Touch		
	Homo neanderthalensis skull model*	Touch		
	Homo sapiens skull model*	Touch		
Plants	Fern fossil	Touch	Y	Biodiversity<br><br>Adaptation (to different climates and environments – pollinators, herbivores, fire, etc…)
	Ferns (2 species)	Touch		
	Seeds from 20 different species	Touch		
	8 species of succulents/cacti	Touch		
	Heartleaf Philodendron	Touch		
	Carnivorous monkey cups	Touch		
	Tillandsias	Touch		
	Cinamon sticks	Touch; Olfaction		
	Ficus bonsai	Touch		
	Vanilla pod	Touch; Olfaction		
	Bamboo canes	Touch; Hearing		
	Lavender	Touch; Olfaction		

	Thyme	Touch; Olfaction		
	Mint	Touch; Olfaction		
	Scented geranium	Touch; Olfaction		
	Cotton	Touch		
	Cork oak bark	Touch		
	Cork oak trunk section	Touch		
	Leaves of different shapes from 4 oak species	Touch		
	Olive tree branch	Touch		
	Olives	Taste		
	Cypress tree	Touch		
	Pine tree	Touch		
	Pine tree trunk section	Touch		
	Pine cones from 10 different species	Touch		
