# Supplementary material for: evALLution: making basic evolution concepts accessible to people with visual impairment through a multisensory tree of life
Source: Evolution (N Y). 2021 Mar 11;14(1):5. doi: 10.1186/s12052-021-00143-1 (PMC7952356; doi:10.1186/s12052-021-00143-1)
Supplement: Supplementary file 4 — Additional file 4. Description of the branch exercises applied to assess prediction ability. [file 12052_2021_143_MOESM4_ESM.docx]

**Scripted branch-specific exercises**

*All questions were used to calculate the branch-specific activity score*

*Questions used for calculating prediction score are marked with (*)*

**Taxon: Plants**

On the evolution of plant roots and adaptation to the terrestrial habitat

*Q: Were the characteristics necessary for plants to conquer land already existing in a few aquatic plants or did they emerge after the plants arrived on land?

On plant adaptations to different climates and pollinators

*Q: What do you think would happen if:

Q.1: …Several pollinators would disappear from the environment?
 Q.2: …The climate changes and becomes very warm in a pine tree forest region?

Q.3: …The climate changes and fires become frequent in a forest of corak oak?

**Taxon: Corals**

On coral sensitivity to environmental change

*Q: what do you think will happen in the water temperature rises?

*Q: Do you think corals can adapt to such an environmental change like temperature rise?

*Q: and do you think that if that environmental change is slow the polips have higher probability of adapt and survive?

**Taxon: Fish**

On the environment-specific lateral plate phenotypes of stickleback fish

*Q: Do you know or can you imagine a reason behind the fact that marine stickleback have a fully armoured body while freshwater ones don’t?

*Q: Do you think stickleback encounter more predators on the marine or freshwater environments?

*Q: which characteristics did you use to answer this question?

*Q: Knowing that baby stickleback are easier to predate upon and that in freshwater their main predators are small dragonfly larvae, in which environment do you think stickleback develop faster?

**Taxon: Mollusks**

Upon touching the shell diversity

*Q: Do you think this shell diversity is somehow associated to different environments?

In a environment with a lot of shell predators, natural selection pressure will favor those with defense strategies

*Q: Which one of these do you think would be more predated upon (smooth cone snail v.s. spiny sea snal)?

*Q: What if I told you that the smooth cone snail is highly venomous? Which strategy would be more effective? Does it depend on the predator or not?

Q: if I tell you that the smooth and venomous snail lives 2 years and leaves a descent of 5 baby snails that survive to adulthood; and the spiny snail lives 5 years (more than the smooth) and leaves a descent of 20 baby snails, but only 2 survive, which one do you think is better adapted?

Q: do you think the development of these different adaptations is a fast or slow process?

Q: And considering all the diversity of mollusks, do you think they exist for long on planet earth?

**Taxon: Homo sapiens**

*Q: Taking into account that features of our species that suffered a lot of alternations in the last million years are related with our jaws and teeth, what do you think might have changed in the environment that might have selected for our current characteristics?

**Taxon: Turtles**

*Q: Can you imagine which advantage might be in having a structure like a turtle shell?

Q: How does such a structure (turtle shell) evolves? Does it happen randomly? Does it confer some advantage to explore the environment?

Q: If ancestor turtles with stronger shells survive more, what happens with time?

*Q: If humans heavily predate on turtles with big and ornate shells, which turtles will survive more in the long term? Those with these desirable characteristics, or those with thinner shells that don’t attract the predator as much?

Q: does the shell remain as an advantageous characteristic in this case?

*Q: Can this mean that turtles will evolve to lose their shell?

*Q: What if besides predating on the turtles for shells, we also steal turltes eggs for food? Can they adapt to several predatory pressures at the same time?

**Taxon: Beetles**

Q: Do you think the beetle’s horns have always been like the one you are feeling, since forever or did they evolved with time?

Q: Do you think that the horns evolved by chance or because those structures conferred some advantage to males bearing them?

**Taxon: Birds**

After feeling the diversity of beak and the respective food sources

Q: If the food source that these birds are adapted to disappear form the environment, what do you think would happen?
