# Supplementary material for: evALLution: making basic evolution concepts accessible to people with visual impairment through a multisensory tree of life
Source: Evolution (N Y). 2021 Mar 11;14(1):5. doi: 10.1186/s12052-021-00143-1 (PMC7952356; doi:10.1186/s12052-021-00143-1)
Supplement: Supplementary file 5 — Additional file 5. Questionnaire for data collection applied to participants before and after the MSToL experience. [file 12052_2021_143_MOESM5_ESM.docx]

**BEFORE THE ACTIVITY**

* AC stands for ‘answer code’, which corresponds to the data recorded on Table S2, used for analysis

1. Age:
2. Gender: M / F
3. Education level:
4. (Academic) Biology Knowledge? **Y/N** (AC*: 1/0)
5. (Academic) Evolution Knowledge? **Y/N** (AC*: 1/0)
6. Nature Interest:

**I don’t have any interest** (AC*: 1)

**I have some interest** (If I catch a documentary on tv/youtube I’ll listen) (AC*: 2)

**I have a lot of interest** (I search for documentaries and books on the subject) (AC*: 3)

1. How frequently do you try to be outside? (AC* scale from 1= Never to 7= Every day)

**Never; Rarely; 1x month; >1x month; 1x week; >1x week; Every day**

1. How frequently do you have contact with animals? (AC* scale from 1= Never to 7= Every day)

**Never; Rarely; 1x month; >1x month; 1x week; >1x week; Every day**

1. Interest in Evolution

**I don’t have any interest** (AC*: 1)

**I have some interest** (If I catch a documentary on tv/youtube I’ll listen) (AC*: 2)

**I have a lot of interest** (I search for documentaries and books on the subject) (AC*: 3)

1. Do you have visual disability? **Y/N** (N includes people with glasses correcting for normal vision levels) (AC*: 1/0)
2. Since when do you have visual disability (**in years**)? _______
3. What’s your level of visual disability?
   1. **Moderated** low vision with perception of shapes and colors (can have lenses) (AC*: 1)
   2. **Deep with residual light perception** (AC*: 2)
   3. **Profound without any light/visual cue perception** (blind) (AC*: 3)
4. Have you done activities in this room before? **Y / N** (AC*: 1/0)
5. From the following words, which ones do you associate with the concept of **Evolution** (circle the ones the participant indicates positively)
   *colors follow the codes on Fig.4 and Fig.S5, see methods for description of word categories.

Perfecting; chance; mutation; progress; species; modification; fossil; ancestral; ramification; extinction; tree; adaptation; descendants; biodiversity; reproduction; environment; emergence; ecology; nature; human; natural selection; diversity; artificial selection; Darwin; dinosaur; behavior; change; science; speciation; common ancestor; development; origin; survival

1. Classify as **True or False**
2. Living organisms have always existed as we know them nowadays **T F**
3. An animal which is better adapted to its environment has more descendants that survive **T F**
4. Currently living organism are adapted to their environments and so they don’t evolve anymore **T F**
5. Humans have common ancestors with starfish and bacteria **T F**
6. Humans evolve differently from plants and animals **T F**
7. Plants evolve less than animals because they don’t move **T F**
8. Mammals are more evolved than fish **T F**
9. Vision is the sense more used by animals **T F**
10. All living beings on earth live independently of one another **T F**

**AFTER THE ACTIVITY**

* AC stands for ‘answer code’, which corresponds to the data recorded on Table S2, used for analysis

**If the participant has visual disability:**

(If not, jump to question 6)

1. Did you use a mobility assistant? **Cane** (AC*: 1)**; Dog** (AC*: 2)**; Family/friends** (AC*: 3)**; Staff evALLution** (AC*: 4)

2. Do you usually use this mobility assistant on your daily routine? **Y / N** (AC*: 1/0)

1. How independent did you feel during the exploration of the Tree-of-Life?
   (AC* scale from 1= Totally to 7= Nothing)

**Totally; A lot; A little; Nothing**

1. How confortable did you feel during the exploration of the Tree-of-Life?

(AC* scale from 1= Totally to 7= Nothing)

**Totally; A lot; A little; Nothing**

1. Did the carpet provide enough texture for you to be able to guide yourself by the floor pattern?

(AC* scale from 1= Totally to 7= Nothing)

**Totally; A lot; A little; Nothing**

1. Which sense did you find most informative? **Vision** (AC*: 1) **Touch** (AC*: 2) **Olfaction** (AC*: 3) **Hearing** (AC*: 4) **Gustation** (AC*: 5)
2. Which sense did you find least informative? **Vision** (AC*: 1) **Touch** (AC*: 2) **Olfaction** (AC*: 3) **Hearing** (AC*: 4) **Gustation** (AC*: 5)
3. Which was your **favorite** tree-of-life branch? _________
4. From the following words, which ones do you associate with the concept of **Evolution** (circle the ones the participant indicates positively)
   *colors follow the codes on Fig.4 and Fig.S5, see methods for description of word categories.

Perfecting; chance; mutation; progress; species; modification; fossil; ancestral; ramification; extinction; tree; adaptation; descendants; biodiversity; reproduction; environment; emergence; ecology; nature; human; natural selection; diversity; artificial selection; Darwin; dinosaur; behavior; change; science; speciation; common ancestor; development; origin; survival

1. Classify as **True or False**
2. Living organisms have always existed as we know them nowadays **T F**
3. An animal which is better adapted to its environment has more descendants that survive **T F**
4. Currently living organism are adapted to their environments and so they don’t evolve anymore **T F**
5. Humans have common ancestors with starfish and bacteria **T F**
6. Humans evolve differently from plants and animals **T F**
7. Plants evolve less than animals because they don’t move **T F**
8. Mammals are more evolved than fish **T F**
9. Vision is the sense more used by animals **T F**
10. All living beings on earth live independently of one another **T F**
