# Supplementary material for: evALLution: making basic evolution concepts accessible to people with visual impairment through a multisensory tree of life
Source: Evolution (N Y). 2021 Mar 11;14(1):5. doi: 10.1186/s12052-021-00143-1 (PMC7952356; doi:10.1186/s12052-021-00143-1)
Supplement: Supplementary file 6 — Additional file 6. Additional figures and data visualization. [file 12052_2021_143_MOESM6_ESM.docx]

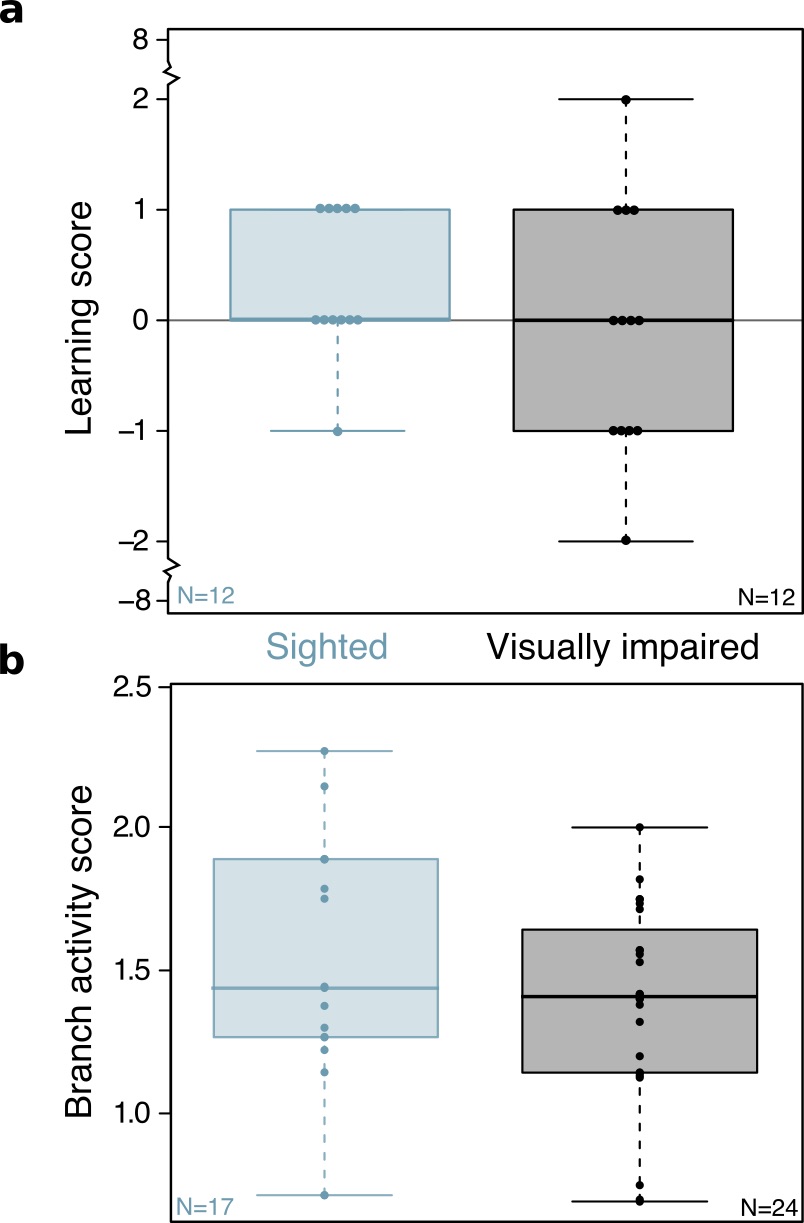


**Figure S2. Participant scores for true or false questionnaire and branch-specific activities assessing basic concepts of evolution.** Score is standardized for number of answers and reflects the total number of correct answers. Learning score was calculated as the difference between the score of a participant after and before the activity (see methods section for further details on score calculation). Sampling numbers for each group are shown under respective boxplots. There are no differences between the two groups of participants (Man-Whitney u test: p-value= 0.275).


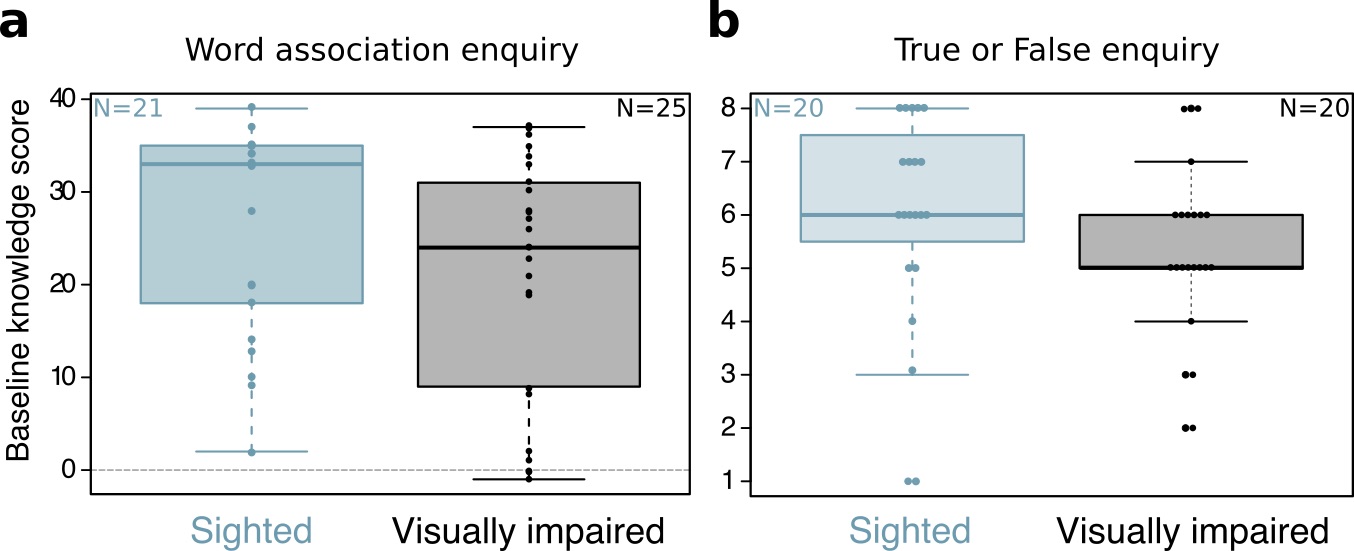


**Fig S3. Participant baseline evolution knowledge on data collected before experiencing the MSToL**. Score is calculated by summing the number of correct answers; sampling numbers for each group are shown for sighted (blue) and visually impaired (black) participants. There are no differences between the two groups of participants for their baseline knowledge measured by the word association exercise (Man-Whitney u test: p-value= 0.16) or by the true or false enquiry (Man-Whitney u test: p-value= 0.065).


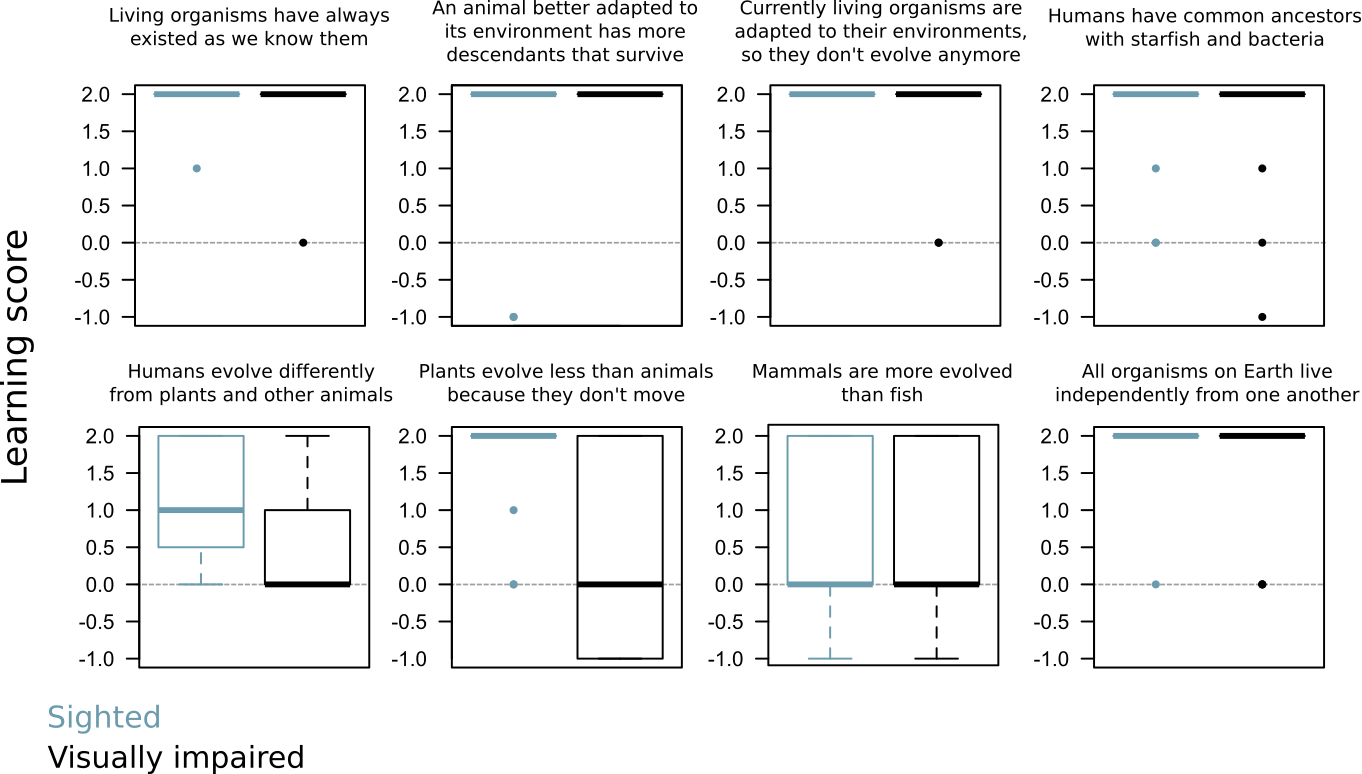


**Figure S4. Learning scores *per* individual question.** These true or false questions were asked to participants before and after the activity and learning score is calculated as the difference between the number of correct answers before and after experiencing the MSToL. The dashed grey line marks the zero: reflecting no change in the score before and after the activity. There are no differences between the two groups of participants (Shapiro test for normality showed none of the datasets was normally distributed and Man-Whitney u tests revealed no differences between sighted and visually impaired participants for any individual question; see code file for individual statistics code and results).


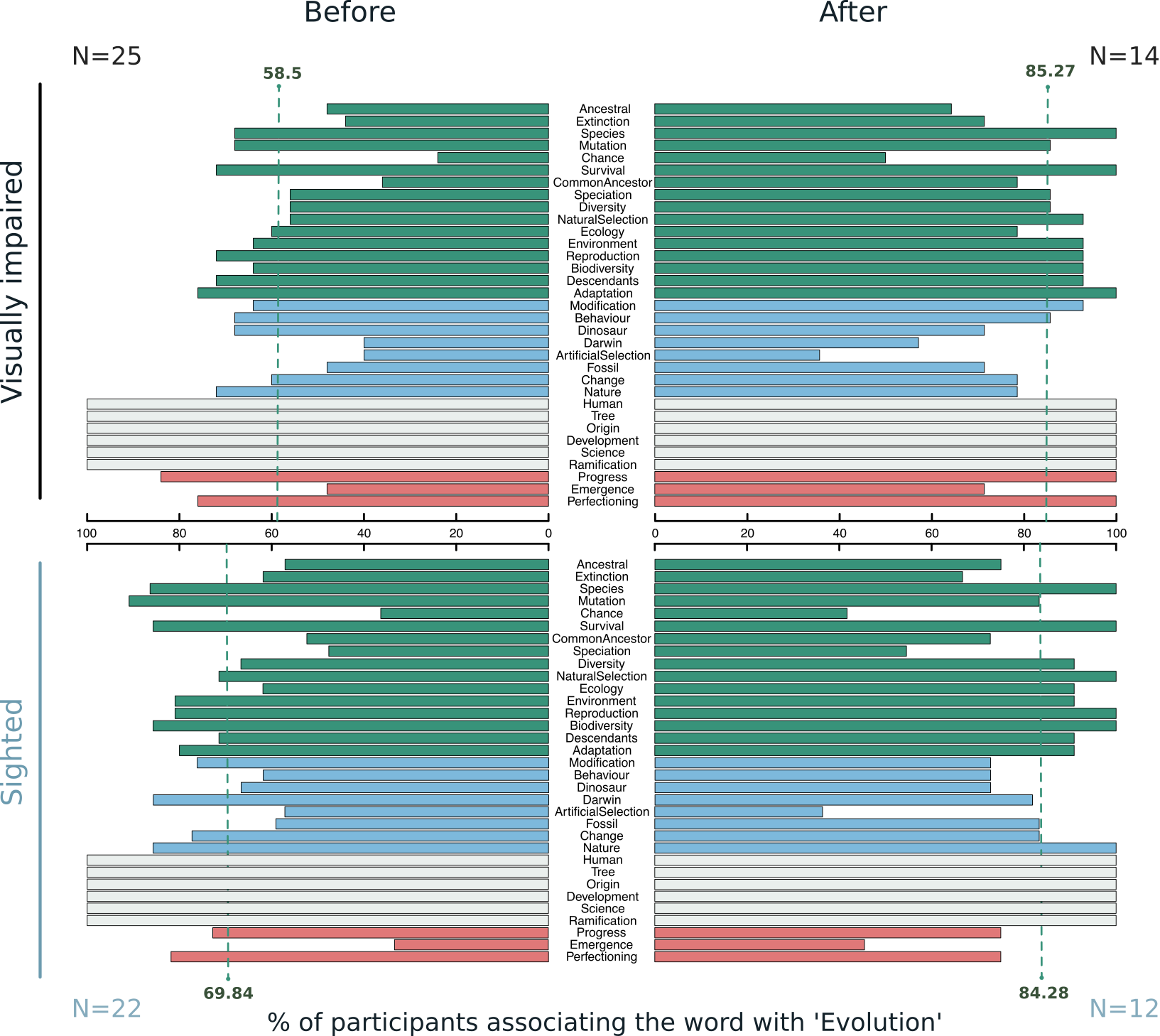


**Figure S5. Visualization of the word association exercise before and after experiencing the MSToL.** Each bar show the percentage of participants associating the word read to them by the volunteer data collector with the concept of ‘evolution’. Plotting settings follow those described in detail on Figure 4.
