# Supplementary material for: evALLution: making basic evolution concepts accessible to people with visual impairment through a multisensory tree of life
Source: Evolution (N Y). 2021 Mar 11;14(1):5. doi: 10.1186/s12052-021-00143-1 (PMC7952356; doi:10.1186/s12052-021-00143-1)
Supplement: Supplementary file 7 — Additional file 7. Inclusive description of the publication figures for people with visual disabilities. [file 12052_2021_143_MOESM7_ESM.pdf]

Accessible description of the figures in Laurentino et al., 'evALLution: making basic evolution concepts accessible to people with visual impairment through a multisensory tree of life'.

Figure 1: A scheme composed of text and photos organized in three lines and three columns titled 'Basic framework to construct evolution outreach activities inclusive for people with blindness'. The first column contains the text list describing the three key steps for the reproduction of the multisensory tree of life and the second and third columns respectively have photos that illustrate the steps described on the first column, both with collections easily obtainable (Mollusks), and harder to obtain referring a classic text-book example of evolution (Tanganyika's cichlid fishes). Step 1: 'Represent biodiversity in an inclusive way'. Collect as many different specimens as possible and make sure they can be touched. In the same line, column two shows a photo of about 40 mollusk shells of gastropods (spiraling sea snail shells) and bivalvia (double valved sea shells) of very different shapes, sizes and colors (white and beige and tons of brown being the dominant ones, but also some green, yellow and orange ones). The shells are haphazardly distributed on a tabletop. Column three shows a collection of 16 dried fish, of different sizes and shapes, mainly dark in color, haphazardly distributed on a tabletop. Step 2: 'Give evolutionary context'. Display specimens according phylogenetic patterns, or display specimens regarding environmental information. Referring to this step, column two shows a photo of the diverse mollusk shells but now organized according to a basic phylogeny that divides the shells into three branches: monoplacophora, bivalvia and gastropoda. From the single phylogeny root (representing the common ancestor of all those mollusks), monoplacophora (single shelled mollusks) branch out first, and then the remaining branch divided into two other, one for bivalvia and another for gastropoda (Notice that this phylogenetic display may be deprecated meanwhile, as these change with new genetic and morphologic information). The before haphazardly distributed shells have now been given evolutionary context by organizing them according to the known phylogenetic relationships. A pattern resulting from evolution and speciation (branching). Column three shows the previously haphazardly distributed fish now organized in different containers across the table: bottom dwelling fish are in shallow containers with sand, closer to the edge of the table which is lined by stones to mimic the lake floor. Surface-dwelling fish are on high stalked glass containers, with water, further way from the table edge, away from the participant. This intended to give the participant the idea that the different

species and body shapes, in nature, are adapted to different depths, and food niches, in the lake. Thus giving the participant ecological information to relate with the morphological adaptations they could feel. Step 3: 'Discuss the process'. Introduce participants to basic evolution concepts, teach the participants to read phylogenies and perform predictive exercises to understand if the public can predict adaptive phenotypic change in response to environmental change. This step refers to the within-branch activities developed, such as the one on column two, where the photo of the mollusk phylogenetic display previously described now shows a participant placing a fossil on the phylogeny branch based on the morphological characteristics of the fossil. This is to introduce the participants to shared characters and common ancestry. On column three, there is the picture of a participant's hands handling a laterally compressed cichlid fish, with thick and resistant scales, and hinging him between the rocks at the table edge. Indeed this is mimicking a natural behavior of this species (*Altolamprologus compressiceps),* which hinges itself between rocks to escape predators. This then allows us to play a game of prediction of phenotypic changes based on predator-prey interactions and thus discuss adaptation.

By applying all key steps, the public should then have access to evolution patterns (Biodiversity and the relationship between species, phylogenies and environments) and evolution processes that give rise to those patterns (mutation, adaptation, natural selection, speciation, etc.).

Figure 2: A figure composed of two photos of the activity displayed in one column, two lines; and, to the right of the photos, a blueprint of the room with the multi-sensorial tree of life; titled 'The multisensory tree-of-life'. **Panel a** consists of the two photos. On the top photo: a top overall view from the ongoing activity, inside the multisensory tree of life room, from above the entrance door. Close to the walls of the room one can see the tables with the tactile materials on top, aligned along the phylogeny branch tips. In each branch a volunteer educator ready to provide guidance and auditory information to each participant. We can see around 16 people scattered inside the room, and a blind participant with his guide dog (laying relaxed on the floor) in the forefront, talking to the volunteer educator of the Fungi branch. All tables are covered in white paper. There's a lot of light in the room and the dark brown carpet phylogeny is stuck on the floor, contrasting clearly with the white walls and the light brown floors.

On the other photo: a top overall view from the tree of life room from the wall directly opposite to the entrance door. The same overall aspects are shared with

the photo described before, but now there are about 30 people in the room, with several participants distributed across branches. The bird branch table appears in the in the forefront, where a blind participant is feeling materials, while holding her guiding cane too. Participants are seen in conversation with the educator volunteers; **Panel b** consists of the blue print, which represents a top view from the room where the outreach activity took place. A 125m^2 room with entrance on the right. On the floor, occupying the majority of the room area, there is the basic scheme of the tree of life topology, drawn with carpet. There are 21 branches distributed along the saloon walls. On each branch tip there is the scheme of a table containing specimens representative of its specific taxonomic group. Starting at the entrance door and following the phylogeny from its root, a participant would find, first, to their right the bacteria branch, then to the left, the plants, then, again to the right, the fungi, then the corals, the echinoderms, the arthropods, the mollusks (these two being the most diverse branches of the eukaryote tree of life), then the cartilaginous fish, then amphibians, bony fish, reptiles, birds, and mammals. At each branch tip, the participant could find a table with mainly real specimens of pedagogic collections and feel the real textures and shapes found in nature. Every branch tip refers to extant taxa but in some branches, on the way to the branch tips, the participants could find extinct taxa, like the archaeopteryx at the mind-branch of birds, a fern fossil at the mid-branch of plants, and the majority of *Homo* ancestors (skull replicas based on fossil evidence) along our evolution branch.

Figure 3: A figure composed of three graphics aligned in a single column titled 'Participant education data and scores based on basic knowledge of evolution before and after the activity, and prediction of evolution outcomes'. **Graphic a**: Depicts a scatter plot with trend lines on the relationship between participant's age and education level. In the xx axis there is age of the participants (which ranges from 19 to 90) and on the yy axis there is Education level (which ranges from 1: primary school, to 5: Master degree). Lines follow the linear model per participant category and vertical dashed lines mark the average age of each group (58 for sigthed and 62 for people with visual impairment). The pattern shows that for similar average age, sighted people tend to have reached higher levels of education. A pattern reflecting the difficulties of education accessibility for people with visual disability; **Graphic b**: Depicts a scatter plot with trend lines - regression analysis with 'Score after the activity' dependent on 'Score before the activity. Participants' score (ranging from 0 to 8) before (xx axis), and after (yy axis) the activity. Each dot refers to a single participants coded black for people with visual

impairment and blue for sighted people. The data refers to the scores of true or false questions on basic evolution concepts applied before and after the MSToL activities where lines show the correlation between before and after score. 12 sighted participants and 13 participants with visual impairment were interviewed. The trends are very similar for both groups (r squared is 0.8 for sighted and 0.62 for visual impaired) but the scatter of people with visual impairment is wider and comprises the lower scores registered, while the scatter of sighted participants is closer to the 0 line and includes de higher scores registered; **Graphic c**: is titled "Evolution prediction" and depicts a boxplot, where the boxes for normovisual and people with visual deficiency stand side by side with the value for "prediction score" on the yy axis. The data is based on participants' predictive ability in scenarios where environments shape phenotypic responses. The average of the evolutionary outcome prediction is slightly higher for sighted (1.42) people, comparative to people with visuall impairment (1.33) but the data distribution is quite similar between the two groups. 17 sighted participants and 23 participants with visual impairment were interviewed.

Figure 4: A figure composed of two horizontal barplots aligned in one row and two columns titled 'Terms associated with the concept of evolution before and after the MSToL activity'. On the xx axis there is the difference of the frequency of the word association and along the yy axis each of the words (the delta score of the word association, meaning that if the word decreased in association after the activity, the bar will depict a negative value, it will be at 0 if there was no change in the association of the word, and have positive value if the association of the word increased after the activity). The graph on the left refers to the sighted group of participants, and the one on the right to the participants with visual impairment. The list of 33 words are depicted along the yy axis, with a bar *per* word, from top to bottom, following a color code, as follows: at the top, in green, words necessary and fundamental to explain and understand evolution (*ancestral, survival, species, speciation, natural selection, mutation, extinction, diversity, common ancestor, chance, ecology, environment, reproduction, biodiversity, descendants, adaptation*); next, in blue, words not necessary to explain the theory of evolution but related to it (*modification, behavior, dinosaur, Darwin, artificial selection, fossil, change, nature*); next, in grey, neutral words and unnecessary to explain the theory of evolution (*human, tree, origin, development, science, ramification*); finally, at the bottom, in red, words usually associated with misunderstanding of the evolutionary process (*progress, emergence, perfectioning*). The overall pattern is similar

between sighted participants and those with visual impairment, in the sense that the majority of the terms increased in their association with the concept of evolution. However, for sighted people, more terms decreased (depicting negative value bars): *mutation, modification, Darwin, artificial selection* and *perfectioning*; and for people with visual impairment, *artificial selection* was the only word showing decreased association and in general all terms increased in association more, compared to sighted participants. In both groups, the association of neutral (grey) words remained the same.

Figure 5: A figure composed by three pie charts (donut charts, as they are hollow in the center, where one can read the respective question asked to the volunteer educators) aligned in two lines and two columns titled ‘Volunteer educator emotional experience’. 15 of the volunteer educators participated in the survey. The plots are coded by color, explained bellow for every plot. By the dominant color and area occupied on the graphic ring we perceive the overall patterns of volunteer educators’ experiences. **Graphic a** where light grey corresponds to answer ‘Visually impaired under 60 years old’, dark grey to ‘Visually impaired above 60 years old’, light blue to ‘Sighted children 5 to 7 years old’, dark blue to ‘Sighted children 7 to 10 years old’, light green to ‘Sighted adults above 60 years old’, and dark green to ‘Sighted adults under 60 years old’. This donut plot refers to the question ‘As an educator, I felt more at ease communicating with…’ and the majority of educators chose ‘Sighted children 7 to 10 years old’, with 5 educators answering this; and ‘Visually impaired above 60 years old’, also 5 educators answering this. 3 educators chose ‘Visually impaired under 60 years old’, 1 educator chose ‘Sighted adults under 60 years old’ and another chose ‘Sighted children 5 to 7 years old’; In **Graphic b,** light green to ‘Nothing’, and dark green to ‘A little’, light grey corresponds to answer ‘Moderately’, light blue to ‘A lot’, dark blue to ‘Immensely’. This donut plot refers to the question ‘As an educator, I learned…’ to which the majority (8) of educators answered ‘Immensely’, 5 answered ‘A lot’, 1 answered ‘Moderately’, and no one answered wither ‘A little’ or ‘Nothing’; **Graphic c** follows the same color scheme described for Graphic b, but this one asks ‘As an educator I had fun…’ to which the majority of educators answered either ‘Immensely’ (7 educators) or ‘A lot’ (also 7 educators) and 1 educator answered ‘Moderately’, with no one answering ‘A little’ or ‘Nothing’. These data allows us to assess the sighted experience of educators who were mainly inexperienced in outreach with people with visual impairment.
